# The *miR-372*-ZBTB7A Oncogenic Axis Suppresses TRAIL-R2 Associated Drug Sensitivity in Oral Carcinoma

**DOI:** 10.3389/fonc.2020.00047

**Published:** 2020-01-31

**Authors:** Li-Yin Yeh, Cheng-Chieh Yang, Hsiao-Li Wu, Shou-Yen Kao, Chung-Ji Liu, Yi-Fen Chen, Shu-Chun Lin, Kuo-Wei Chang

**Affiliations:** ^1^Department of Dentistry, School of Dentistry, Institute of Oral Biology, National Yang-Ming University, Taipei, Taiwan; ^2^Department of Dentistry, National Yang-Ming University, Taipei, Taiwan; ^3^Department of Stomatology, Taipei Veterans General Hospital, Taipei, Taiwan; ^4^Department of Dentistry, MacKay Memorial Hospital, Taipei, Taiwan

**Keywords:** apoptosis, *miR-372*, suppressor, ZBTB7A, TRAIL-R2

## Abstract

*miR-372* has been shown a potent oncogenic miRNA in the pathogenesis of oral squamous cell carcinoma (OSCC). The zinc finger and BTB domain containing 7A protein (ZBTB7A) is a transcriptional regulator that is involved in a great diversity of physiological and oncogenic regulation. However, the modulation of ZBTB7A in OSCC remains unclear. Tissue analysis identifies a reverse correlation in expression between *miR-372* and ZBTB7A in OSCC tumors. When OSCC cells have stable knockdown of ZBTB7A, their oncogenic potential and drug resistance is increased. By way of contrast, such an increase is attenuated by expression of ZBTB7A. Screening and validation confirms that ZBTB7A is able to modulate expression of the death receptors TRAIL-R1, TRAIL-R2, Fas and p53 phosphorylated at serine-15. In addition, ZBTB7A transactivates TRAIL-R2, which sensitizes cells to cisplatin-induced apoptosis. The ZBTB7A-TRAIL-R2 cascade is involved in both the extrinsic and intrinsic cisplatin-induced pathways of apoptosis. Database analysis indicates that the expression level of and the copy status of ZBTB7A and TRAIL-R2 are important survival predictors for head and neck cancers. Collectively, this study indicates the importance of the *miR-372*-ZBTB7A-TRAIL-R2 axis in mediating OSCC pathogenesis and in controlling OSCC drug resistance. Therefore, silencing *miR-372* and/or upregulating ZBTB7A would seem to be promising strategies for enhancing the sensitivity of OSCC to cisplatin therapy.

## Introduction

Head and neck squamous cell carcinoma (HNSCC), including oral SCC (OSCC), is one of the prevalent neoplasms worldwide (1, 2). When develop new strategies that block HNSCC development, a molecular understanding of HNSCC pathogenesis is important (1). Over the last decade, many studies have indicated that miRNAs and theirs targeted genes are highly associated with OSCC promotion and progression (3–9).

The miRNAs *miR-371, miR-372*, and *miR-373* form a miRNA cluster on chromosome 19q13, a locus where many oncogenic events related to HNSCC are known to reside (10). This cluster of miRNAs was originally found to be crucial to the maintenance of stemness in embryonic cells (11). *miR-372/miR-373* were then found to be oncogenes that target LATS2, CD44 and various other differentiation regulators active in tumors (12, 13). They are upregulated in malignancies and their upregulation of expression of *miR-372*/*miR-373* has been found in HNSCC and *miR-372* expression in tumors is a prognostic marker of OSCC (6, 8, 14). Serum *miR-372* levels are potential diagnosis and prognosis biomarkers in neoplasms including HNSCC (4, 15). In addition, *miR-372* expression is hypoxia inducible, and such induction can then result in a repression of RECK in OSCC (5). Furthermore, we have identified previously that *miR-372* targets p62, which, in turn, enhances OSCC cell progression (4).

The Zinc finger and BTB domain containing 7A protein (ZBTB7A, also named Pokemon, FBI or LRF in various articles) belongs to the POK (POZ/BTB domain and Krüppel-type zinc finger) family of transcriptional regulators and resides at chromosome 19p.13.3 (16). This protein binds to GC-rich sequences in promoters and then interacts with various cofactors via its POZ domain (17). ZBTB7A is a pleotropic transcription factor implicated in multiple physiological or pathological processes (18). It has been regarded as proto-oncogene due to its ability to repress various tumor suppressors including ARF (19). However, studies also found that ZBTB7A may also interact with and repress SOX9 (sex determining region Y-box 9), various glycolytic transcription factors and a number of other targets; these findings reveal this protein's functional complexity when mediating tumor suppression (16, 17, 19–22). Although the roles of ZBTB7A in carcinogenesis are controversial and the mechanisms by which it acts remain largely obscure, frequent deletion and downregulation of ZBTB7A has been shown to occur in a range of malignancies including OSCC (20, 23–25). In addition, *miR-106b* and other miRNAs have been shown to target ZBTB7A in such malignancies (25–28).

The tumor necrosis factor related apoptosis-inducing ligand (TRAIL) engages with TRAIL receptor (TRAIL-R) family members, such as TRAIL-R1 (DR-4) and TRAIL-R2 (DR-5) to elicit apoptosis. TRAIL also binds to TRAIL-R3 (DcR-1) and TRAIL-R4 (DcR-2), which are TRAIL-R members that lack the complete death domain (29). TRAIL-R family member genes are localized at chromosome 8p21.3 and have a tandem alignment (30). As TRAIL-R1 and TRAIL-R2 are apoptosis triggers that are active specifically in cancer cells rather than healthy cells (31, 32), TRAIL-based therapies have become potential cancer targeting strategies. However, targeting TRAIL has disappointing outcomes because resistance to TRAIL therapy is common in cancers (33–36). Specifically, a previous study has shown that the isoforms of TRAIL-R2 may be involved in driving differential apoptotic induction in lung cancer cells (37). Epithelial-mesenchymal transition (EMT) associated N-cadherin expression has been shown to decrease TRAIL-R2 expression and increase DcR-2 expression in OSCC cell line (38). However, the relationship between TRAIL-associated apoptosis and counteracting drug-resistance in HNSCC/OSCC remains to be elucidated.

Cisplatin (CDDP) is a standard chemotherapeutic drug for locally advanced HNSCC. We demonstrate in this study that ZBTB7A suppressor is a new target of *miR-372* and this protein is able to promote CDDP-induced apoptotic cell death through both the intrinsic and extrinsic death pathways. This implies that TRAIL-R2 trans-activation by ZBTB7A underlies *miR-372* associated anti-apoptosis in OSCC.

## Materials and Methods

### Cell Culture, Reagents, and Phenotypic Assays

The SAS, OC3, OECM1, HSC3, and FaDu OSCC cell lines, 293FT cells, phoenix package cells and the hTERT immortalized normal oral keratinocytes (NOK) that were established in our laboratory, were all cultured as previously described (4, 39, 40). NOK and OC3 cells carry wild-type *p53* sequence, SAS cell exhibits wild-type p53 activity, albeit the truncation of *p53* at the C-terminal. The OECM1, HSC3 and FaDu cell lines harbor mutated *p53* sequence (41). Primary dental pulp cells (DPC) or periodontal ligament cells (PDL) had been previously established, or were newly established with approval from The Institute Review Board (IRB) of Taipei Veterans General Hospital (Approval No. 2017-07-023AC) (42). All cells were authenticated by short tandem repeat analysis (Table S1). Cells were seeded on ultra-low attachment culture plates (Corning, Corning, NY) in order to allow them to form 3-dimensional (3D) spheres. CDDP, taxol, 3-methyladenine (3MA), hydroxyurea, and dimethyloxaloylglycine (DMOG) were purchased from Sigma-Aldrich (St. Louise, MO) (4, 39). EGFR Inhibitor AG1478 was purchased from Abcam (Cambridge, UK), and ferristain-1 (ferroptosis inhibitor) was purchased from Santa Cruz Biotech (Santa Cruz, CA). CDDP-resistant (CDDP-R) and taxol-resistant (taxol-R) SAS cell subclones were established by continuous treatment using drug gradients over 6 months. For the CDDP-R subclone, the cells were initially treated with 5 μM CDDP and eventually were treated with a final concentration of 15 μM. For the taxol-R subclone, the cells were initially treated with 2.5 nM taxol and eventually were treated with a final concentration of 10 nM. The *miR-372* mimic, the mirVana™ *miR-372* inhibitor and the various scramble (Scr) controls were purchased from Applied Biosystems (Foster City, CA). They were optimized for use at 100 or 120 nM during cell treatment. The si-ZBTB7A and si-TRAIL-R2 oligonucleotides, together with the si-scramble (si-Scr) control oligonucleotide, were purchased from Santa Cruz Biotech (Table S2). Analysis of cell growth, the cell cycle, BrdU incorporation, migration, invasion, and anchorage-independent growth ability followed various previously published protocols (4, 7). Unless specified, all other materials were purchased from Sigma-Aldrich.

### qRT-PCR Analysis

Total RNA was extracted from cells using TRIzol reagent (Applied Biosystems). TaqMan assay kits (Applied Biosystems) were used to detect the expression of *miR-372, miR-373, ZBTB7A, TRAIL-R1, TRAIL-R2*, and *Fas* (Table S3). The –ΔΔCt between the experimental and control groups of the tested genes were normalized against either *RNU6B* or *GAPDH*. These results were used to calculate the 2^−ΔΔCt^, values, which represent the fold change in expression.

### RNA Sequencing

Total RNA was subjected to mRNA enrichment, fragmentation, reverse transcription, library construction and sequencing (Genomics Co., Taipei, Taiwan). The transcriptome was then aligned according to the various genes' FPKM (Fragments Per Kb of transcript per Million mapped reads) values in order to detect discrepancies in the transcript levels within the various cells.

### Western Blot Analysis

Western blot analysis followed protocols that we previously established using various primary antibodies (Table S4) and a range of secondary antibodies (Table S5) (4, 9). GAPDH was used as the internal control for Western blotting.

### Tissue Samples

RNA and protein were extracted from primary OSCC tumors and their paired non-cancerous matched tissue samples (NCMT); these were then used for qRT-PCR analysis and Western blot analysis (Table S6). Samples were collected after obtaining written informed consent. The tissue study was approved by IRB committee of Mackay Memorial Hospital with approval numbers 11MMHIS026 and 17MMHIS164.

### Reporter Activity Assay

A sequence segment in the 3′UTR of the ZBTB7A gene was predicted by TargetScan software to encompass three potential *miR-372* binding sites, and this was then amplified by PCR (Table S7). The amplicons were cloned into the pMIR-REPORT reporter vector (Applied Biosystems) to produce a wild-type reporter plasmid designated WT. Three mutant reporter plasmids were then created from the WT by replacing the sequence of the ZBTB7A 3′UTR at nucleotide positions 553-559 (GCACUU), 768-774 (GCACUU), and 911-917 (GCACUUU) with AGGTACC, AGGTACC, and GCTCGAGC to give the Mut 1, Mut 2, and Mut 3 reporters, respectively (Table S7). KpnI and XhoI restriction enzyme digestion sites were generated in these mutant reporters by the changes and these sites were used to confirm the successful establishment of the constructs. Firefly luciferase activity normalized against renilla luciferase activity, which represents the transfection efficiency, was used to measure reporter activity.

### Establishment of Constructs and Cell Subclones

A lentiviral vector containing the coding sequence of ZBTB7A and tagged mGFP (Cat. No. RC222759L2) was purchased from OriGene (Rockville, MD). Cell subclones with stable ZBTB7A expression were established by viral infection and GFP sorting. Short hairpin sh-ZBTB7A constructs packed in lentiviruses were purchased from the National RNAi consortium (Academia Sinica, Taipei, Taiwan). Cell subclones exhibiting stable knockdown of ZBTB7A, designated sh-ZBTB7A (6851), and sh-ZBTB7A (7332), together with a sh-Luc control, were achieved by viral infection and puromycin selection (Table S8). Both long form and short form TRAIL-R2 coding sequences were amplified by PCR (Table S7) and sequenced to confirm that they had the correct sequence. These were then cloned into the pBABE-neo retroviral vector (Addgene, Watertown, MA). The stable cell subclones expressing long-form TRAIL-R2 and short-form TRAIL-R2 were obtained by geneticin selection after retroviral infection. To verify the TRAIL-R2 isoforms (40), primers (Table S7) were designed to amplify the long form and the short form transcripts that are the result of differential splicing. Two guide RNAs, 5′sgRNA (CACTATTCTGATGTCCAAG) and 3′sgRNA (GTGACGCCCATATCAACGGA), were designed to knockout mature hsa-*miR-372*-3p (5′-AAAGTGCTGCGACATTTGAGCGT-3′). Each sgRNA was cloned into the pU6-sgRNA.pPuro vector (National RNAi Consortium) and co-transfected with the p5w-Cas9.pBsd vector into cells (7). After the induction of CRISPR activity and selection with puromycin, monoclonal cell subclones were isolated by limiting dilution cultivation. PCR analysis was used to detect gene loss in the subclones (Table S7).

### Apoptosis Array Assay

A Human Apoptosis Antibody Array Kit (ARY009; R&D Systems, Minneapolis, MN) was used to verify the presence of various proteins known to be crucial to apoptosis. Briefly, 250 μg of lysates were prepared and hybridized with an array for 4°C overnight. The array was then washed, which was followed by incubation with an antibody cocktail for 1 h. The signals were detected using a Luminescence/Fluorescence Imaging System (LAS-4000; GE Healthcare Life Sciences; Marlborough, MA).

### Cell Viability and Cell Death Assay

3-(4,5-Dimethylthiazol-2-yl)-2,5-diphenyltetrazolium bromide (MTT) assays were performed to measure cell viability following drug treatment. The results were plotted as dose-response curves. For cell death detection, cells were harvested and stained with PI and annexin V-FITC using an Apoptosis Detection Kit I (Cat. No. 556547; BD Biosciences, Franklin Lakes, NJ). The stained cells were analyzed by CytoFLEX flow cytometry (Beckman Coulter, Palo Alto, CA).

### Mitochondrial Membrane Potential (JC-1) Assay

A MitoScreen Kit (Cat. No. 551302; BD Biosciences) was used to assess the mitochondrial membrane potential (MMP). Cells were stained with JC-1 MMP indicator and analyzed using a CytoFLEX (Beckman Coulter). JC-1 forms monomers at low concentrations and these are measured using the green (FL1) channel. Healthy mitochondria that are significantly polarized undergo rapid uptake of the dye, which then form red fluorescent JC-1 aggregates, which are measured using red (FL2) channel. The red/green ratio of fluorescence represents the health status of the mitochondria within the tested cells.

### Promoter Activity Assay

The promoter regions 2,000-bp upstream of the transcription start sites of *TRAIL-R1* and *TRAIL-R2* gene were cloned into the pGL3-Basic vector (Promega) in order to construct promoter reporter plasmids (Table S7). Constructs were co-transfected with renilla luciferase vector for 48 h. The promoter activity assays were performed using a dual-luciferase reporter assay system (Promega).

### ChIP Assay

An EZ-ChIP™ (Cat. No. 17-371) kit was purchased from Merck (Darmstadt, Germany). Cells were fixed with formaldehyde and processed according to the manufacturer's instructions (7). Amplicons of TRAIL-R2-1 and TRAIL-R2-2 containing putative ZBTB7A binding sites within the *TRAIL-R2* promoter were obtained by amplifying the immunoprecipitated DNA fragments (Table S7).

### Database Analysis

The Targetscan *in silico* module (www.targetscan.org/) was used to predict the target of *miR-372*. The TCGA database (https://portal.gdc.cancer.gov/) was used to determine clinicopathological implications and the patient survival. UCSC Xena (https://xena.ucsc.edu/) and MEXPRESS (https://mexpress.be/) were used to annotate the gene signatures. The Jaspar (http://jaspar.genereg.net) database was used for predicting potential transcription factor-binding sites.

### Subcutaneous Xenografic Tumorigenesis

Nude mice (National Laboratory Animal Center, Taipei, Taiwan) were injected with 5 × 10^5^ various types of SAS cells subcutaneously. After 2 weeks of tumor induction, mice received intraperitoneal injection of CDDP (5 mg/kg) or normal saline three times a week for 3 weeks. Tumor volumes were calculated using the formula: 0.5 × (width)^2^ × length. This animal study was approved by Institutional Animal Care and Use Committee of National Yang-Ming University.

### Statistics

Data are shown as means ± SE. Comparisons were carried out using *t*-tests, two-way ANOVAs and correlation tests. When a *p* < 0.05 it was considered to be statistically significant.

## Results

### Low ZBTB7A Expression in OSCC Is Due to miR-372 Targeting

**Figure 1:**
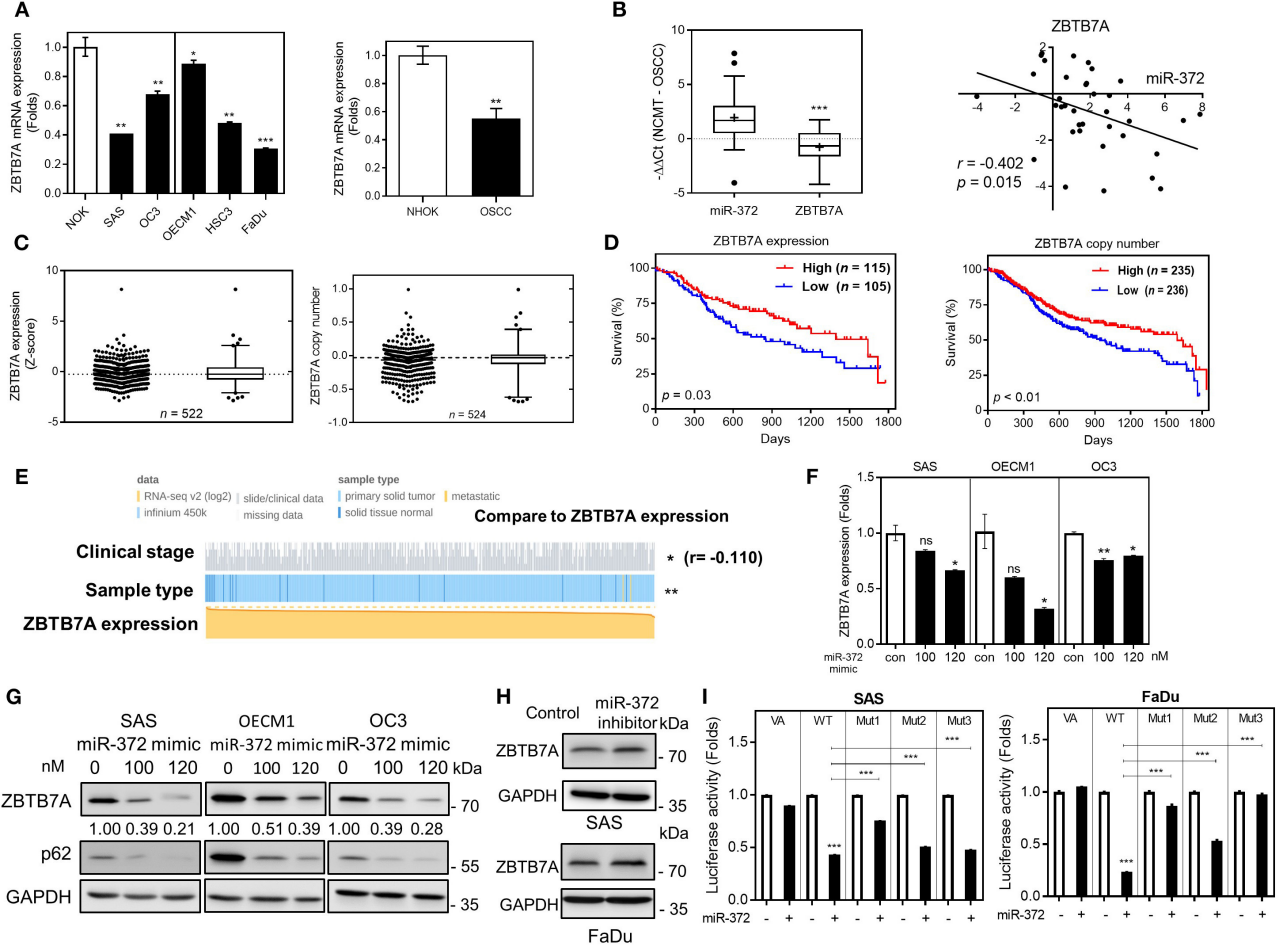
ZBTB7A downregulation in OSCC is due to *miR-372* targeting. **(A)** *ZBTB7A* mRNA expression in OSCC cell lines and normal oral keratinocytes (NOK). Lt panel, individual comparison. Vertical line separates cells having wild-type *p53* (Lt) and mutant *p53* (Rt). Rt panel, overall comparison. **(B)** qRT-PCR analysis of OSCC tissue pairs. Lt, upregulation of *miR-372* expression and downregulation of *ZBTB7A* mRNA expression in OSCC tumors. Rt, correlation analysis showing a reverse correlation between *miR-372* expression (X-axis) and the *ZBTB7A* mRNA expression (Y-axis) in tumors. **(C–E)** Analysis of the TCGA HNSCC database. **(C)** Lt, ZBTB7A expression. Rt, ZBTB7A copy number. In each figure panel, both dot plot (Lt) and Box-and-Whiskers plot (Rt) are shown. Median values marked by dot-lines and are used as cut-offs in order to define high vs. low. **(D)** Kaplan-Meier analysis of patient overall survival according to ZBTB7A expression (Lt) and ZBTB7A copy number (Rt). **(E)** Association between ZBTB7A expression and clinicopathological parameters. ZBTB7A expression is significantly associated with sample origin (normal tissue vs. tumor tissue) and stages. *r* in **(B,E)**, correlation coefficient. **(F,G)** *miR-372* expression downregulates *ZBTB7A* mRNA expression (in **F**) and protein expression (in **G**) in a dose-dependent manner. **(H)** *miR-372* inhibition slightly upregulates ZBTB7A expression in SAS (Upper) and FaDu (Lower) cells. **(I)** Luciferase reporter assay showing the direct targeting of *miR-372* onto the 3'UTR of ZBTB7A (VA, vector alone; WT, wild-type reporter; Mut1–Mut3, mutant reporters). The potential target sequences within the wild-type reporter are replaced by restriction enzyme sites in the three mutant reporters. *ns*, not significant, *p <0.05; **p <0.01; ***p <0.001.

To demonstrate the oncogenic functions of *miR-372* in OSCC, we used the Targetscan platform to predict potential targets of *miR-372*. ZBTB7A was found to be a previously unidentified target of *miR-372*. A comparison with NOK revealed that there was a significant downregulation of ZBTB7A in various OSCC cells lines (Figure 1A). No differential ZBTB7A expression in accordance with *p53* gene state was noted in the cells analyzed. We also compared ZBTB7A expression in DPC14 dental pulp cells and in periodontal ligament cells (PDL9-1, PDL-YM1 and iPDL-2) with expression in SAS cells. ZBTB7A expression was found to be higher in all of the tested normal cells compared to the SAS cells (Figure S1). Furthermore, concordant *miR-372* upregulation and ZBTB7A downregulation could be seen in OSCC tumors (Figure 1B), thus *miR-372* expression and ZBTB7A expression are reversely correlated. In data-sets obtained from TCGA, ZBTB7A expression and ZBTB7A copy number was found to be decreased in HNSCC (Figure 1C). In addition, ZBTB7A downregulation and a decrease in ZBTB7A copy number were found to be associated with a worse HNSCC prognosis (Figure 1D). Further investigation disclosed that there was downregulation of ZBTB7A expression in HNSCC tumors compared to their control normal mucosa, and that the degree of downregulation of ZBTB7A in HNSCC tumors increased in tumors that were at a more advanced stage (Figure 1E). HNSCC tumor stage was also associated with various other parameters (Figure S2). Exogenous *miR-372* expression was shown to dose-dependently decrease ZBTB7A expression at both the protein and the mRNA level, whereas p62, which is a known *miR-372* target, was also found to be downregulated (Figures 1F,G). On the other hand, inhibition of endogenous *miR-372* expression increased ZBTB7A expression (Figure 1H). Three possible *miR-372* target sites within the ZBTB7A 3'UTR were predicted (Figure S3). After transfecting with a *miR-372* mimic, the assays showed that there was a repression of luciferase activity using a WT reporter (Figure 1I). This repression was reversed to different extents when the various mutant reporters were assessed. These findings suggest that ZBTB7A is a novel target of *miR-372* during OSCC.

### ZBTB7A Suppresses miR-372 Associated Oncogenicity in OSCC Cells

**Figure 2:**
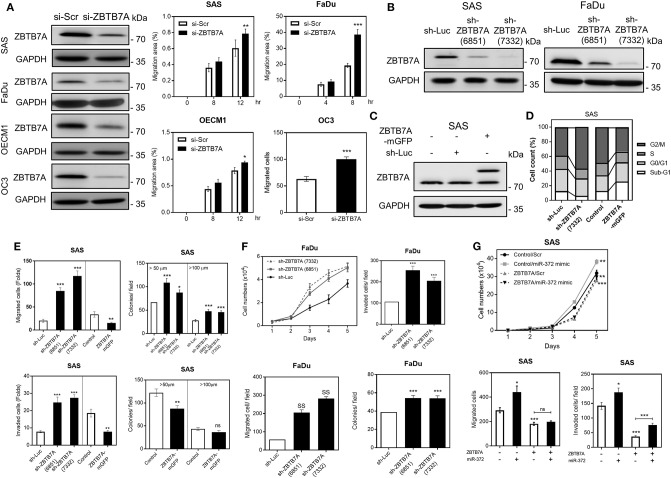
ZBTB7A suppresses OSCC oncogenicity. **(A)** Transient transfection of si-ZBTB7A decreases ZBTB7A protein expression (Lt), and increases cell migration as revealed by wound healing migration assays using OSCC cells (Middle and Rt). Wound closure is used to assess migration ability. **(B,C)** Western blot analysis. **(B)** This reveals the decrease in ZBTB7A expression present in the ZBTB7A knockdown (sh-ZBTB7A) cell subclones. **(C)** The findings also reveal expression of the fusion protein in the ZBTB7A-mGFP cell subclones. **(D)** Summary of cell cycle profiles of the ZBTB7A knockdown and exogenous expression SAS cell subclones. The cell cycle phases in the synchronized cell subclones after CDDP treatment for 24 h are shown. For details, please refer to Figure S4. **(E–G)** Oncogenicity. **(E)** Increases and decreases in oncogenicity can be seen with the ZBTB7A knockdown and exogenous expression SAS cell subclones, respectively. **(F)** Increased proliferation, migration, invasion and anchorage-independent colony formation occur with the ZBTB7A knockdown cell subclone of FaDu. **(G)** *miR-372* associated changes in proliferation (Upper), migration (Lower Lt), and invasion (Lower Rt) of SAS cells are rescued by exogenous ZBTB7A expression. *ns*, not significant, *p < 0.05; **p < 0.01; ***p < 0.001.

OSCC cells displayed ZBTBA downregulation, but the endogenous expression was still present, we performed transient ZBTB7A knockdown, as well as establishing a series of stable knockdown and expression subclones for functional assays. The transient knockdown of ZBTB7A was shown to drastically increase the migration of OSCC cells (Figure 2A). Two stable knockdown subclones, sh-ZBTB7A (6851) and sh-ZBTB7A (7332), were established, and the latter was found to have more conspicuous and effective downregulation in OSCC cells (Figure 2B). A ZBTB7A-mGFP subclone was established in SAS cells (Figure 2C). Analysis of this subclone revealed that the ZBTB7A knockdown increased the number of cells in the G2/M phase of the cell cycle, while decreasing the Sub-G1 fraction. On the other hand, ZBTB7A expression had the opposite effect (Figure 2D; Figure S4). ZBTB7A knockdown in OSCC cells also increased growth, migration, invasion and anchorage-independent colony formation (Figures 2E,F). However, ZBTB7A expression in SAS cells decreased the invasion and colony formation abilities of the cells. To certify that these phenotypes are a result of a *miR-372*-ZBTB7A cascade, the ZBTB7A expression subclone was transfected with *miR-372* mimic. Exogenous expression of *miR-372* resulted in a reversion to lower levels of migration and invasion via control by ZBTB7A (Figure 2G). Thus, the *miR-372* induced oncogenicity of SAS cells is able to be attenuated by ZBTB7A expression.

### ZBTB7A Expression Increases the Drug Sensitivity of OSCC Cells

**Figure 3:**
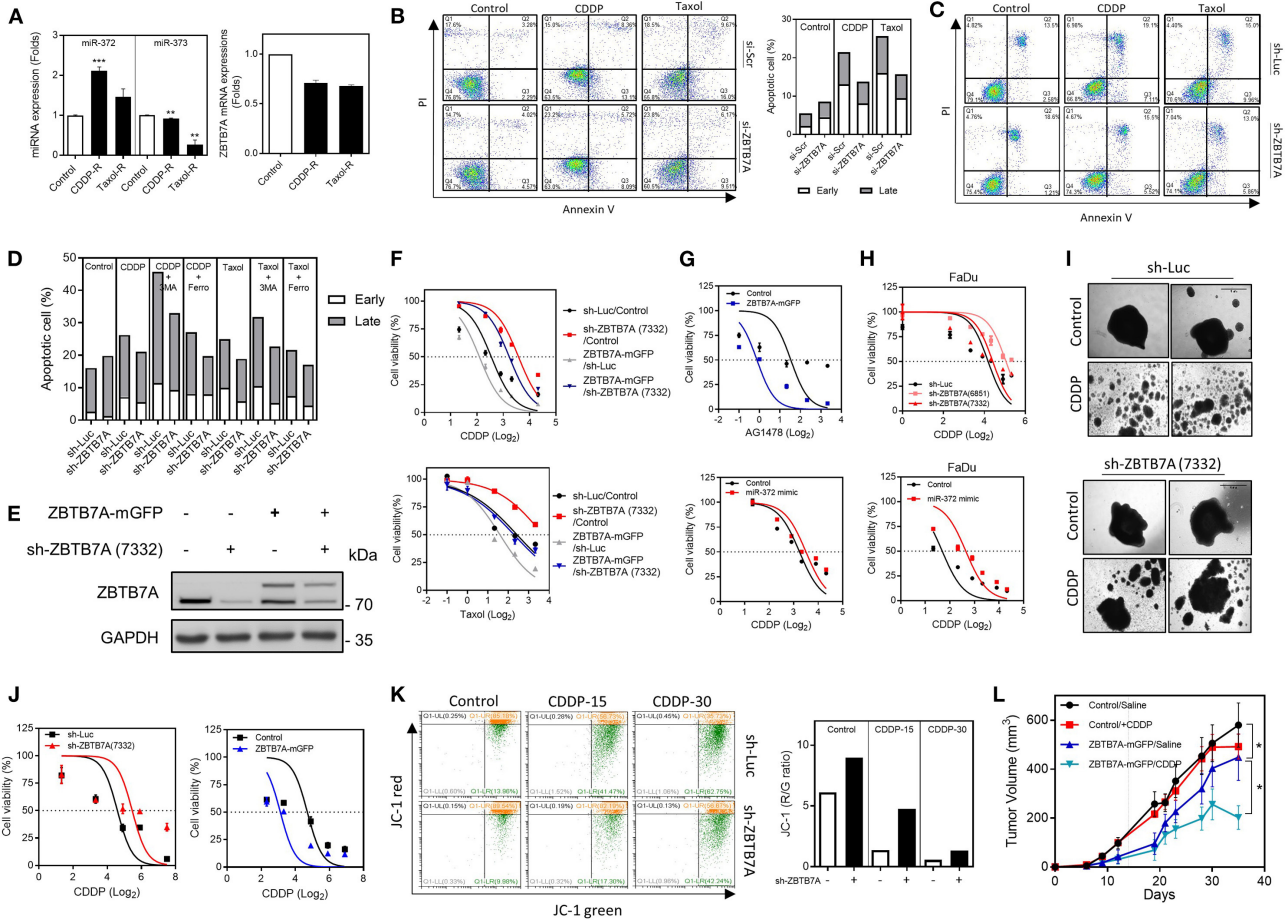
ZBTB7A expression is associated with apoptosis and drug sensitivity in OSCC cells. **(A–G,I–L)** SAS cells. **(H)** FaDu cells. **(A)** Lt, Increased *miR-372* expression in CDDP resistance (CDDP-R) and taxol resistance (taxol-R) SAS cell subclones relative to the parental cells. Rt, ZBTB7A expression is slightly downregulated in these cell subclones. **(B,C)** Apoptosis assay. Cells with transient ZBTB7A knockdown (in **B**, Lt), as well as the stable knockdown cell subclone (in **C**), were treated with CDDP or taxol to induce apoptosis. **(B)** Rt, quantification of the presence of apoptotic cells. Lt, ZBTB7A knockdown decreases the apoptosis induced by the above drugs. **(D)** Quantification of the apoptotic cells. CDDP-induced apoptosis is enhanced by 3MA treatment, but not by Ferrostatin-1 treatment. Taxol-induced apoptosis is slightly enhanced by 3MA treatment, but not by Ferrostatin-1 treatment. **(E)** Western blot analysis. Differential ZBTB7A expression in sh-ZBTB7A (7332) knockdown, ZBTB7A exogenous expression and both knockdown and expression subclones. **(F–H)** Cell viability assays. **(F)** Upper, CDDP. Lower, taxol. These shows an association between ZBTB7A expression level and drug sensitivity. **(G,H)** The results show that ZBTB7A expression sensitizes SAS cells to AG1478 treatment. *miR-372* expression and ZBTB7A knockdown are associated with CDDP resistance in both SAS and FaDu cells. **(I,J)** 3D culture of SAS cells. **(I)** Representative colonies undergoing 3D culture. CDDP disrupts the colonies, while knockdown of ZBTB7A partly restores the integrity of colonies. Bars, 1 mm. **(J)** Quantification of cell viability during 3D culture. Lt, ZBTB7A knockdown. Rt, ZBTB7A exogenous expression. The results show that there is an association between ZBTB7A expression and CDDP sensitivity during 3D culture. **(K)** Mitochondrial membrane potential analysis. Lt, flow cytometry diagrams. The cells were treated with 15 or 30 μM CDDP for 48 h then stained with JC-1. The shift in fluorescence from red to green indicates the collapse of mitochondrial membrane potential. Rt, Quantitative analysis of red/green ratio. CDDP reduces the ratio in a dose-dependent manner, while ZBTB7A knockdown reverses this change. **(L)** Subcutaneous tumorigenicity. ZBTB7A expression decreases xenografic growth, while the CDDP regimen further increases the inhibitory efficacy of ZBTB7A. *ns*, not significant; *p < 0.05; **p < 0.01; ***p < 0.001.

In the CDDP-R and taxol-R cell subclones, *miR-372* was found to be upregulated while ZBTB7A was downregulated in parallel (Figure 3A). Cells were stained with annexin V and PI in order to detect the induction of apoptosis following treatment with either CDDP or taxol. The apoptotic cell fraction was found to be decreased after ZBTB7A downregulation (Figures 3B,C), whereas addition of 3MA, but not ferrostatin-1, enriched the apoptosis induced by CDDP. On the otherhand, 3MA treatment resulted in only a slight increase in taxol-induced cell apoptosis and ferrostatin-1 had no effect on taxol toxicity (Figure 3D). These findings suggest that autophagy, but not ferroptosis, are involved in CDDP induced apoptosis and in taxol induced cell death. Knockdown of ZBTB7A in the ZBTB7A-mGFP subclone by means of sh-ZBTB7A (7332) lentiviral infection was performed using SAS cells (Figure 3E). The sh-ZBTB7A (7332) subclone exhibited the lowest CDDP and taxol sensitivity, while the ZBTB7A-mGFP subclone displayed the highest CDDP and taxol sensitivity. The sensitivity of the sh-ZBTB7A (7332) subclone was increased by ZBTB7A expression (Figure 3F). ZBTB7A expression also sensitized SAS cells to AG1478 treatment (Figure 3G). ZBTB7A knockdown increased the CDDP resistance of FaDu cells (Figure 3H). Furthermore, *miR-372* expression increased the CDDP resistance of both SAS and FaDu cells (Figures 3G,H).

When cells are cultured in a two-dimensional environment, it is well-known that this is not representative of cells in the complex tumor microenvironment. In this context, cells cultured in a 3D environment are known to have a higher resistance to apoptosis than those cultured in monolayer (43). The ZBTB7A knockdown and expression subclones were cultured in ultra-low attachment dishes to allow spheroid formation and then tested for CDDP sensitivity (Figure 3I). Cell viability assays were able to demonstrate that ZBTB7A expression is also associated with CDDP sensitivity in a 3D culture environment (Figure 3J). One possibility is that CDDP is able to induce cytotoxicity via mitochondrial disruption (44). JC-1 staining revealed that there was a reduction in MMP after CDDP treatment and that this occurred in a dose-dependent manner. However, the fluorescence shift from red to green was prevented by ZBTB7A downregulation (Figure 3K). Nine episodes of treatments with a low dose of CDDP only slightly reduced the xenografic growth of SAS cells in nude mice. When there was expression of ZBTB7A in the SAS cells, the CDDP inhibition of these SAS xenografts was significantly increased (Figure 3L). The data support an association between ZBTB7A and drug sensitivity of OSCC cells.

### ZBTB7A Upregulates the Expression of Death Receptors

**Figure 4:**
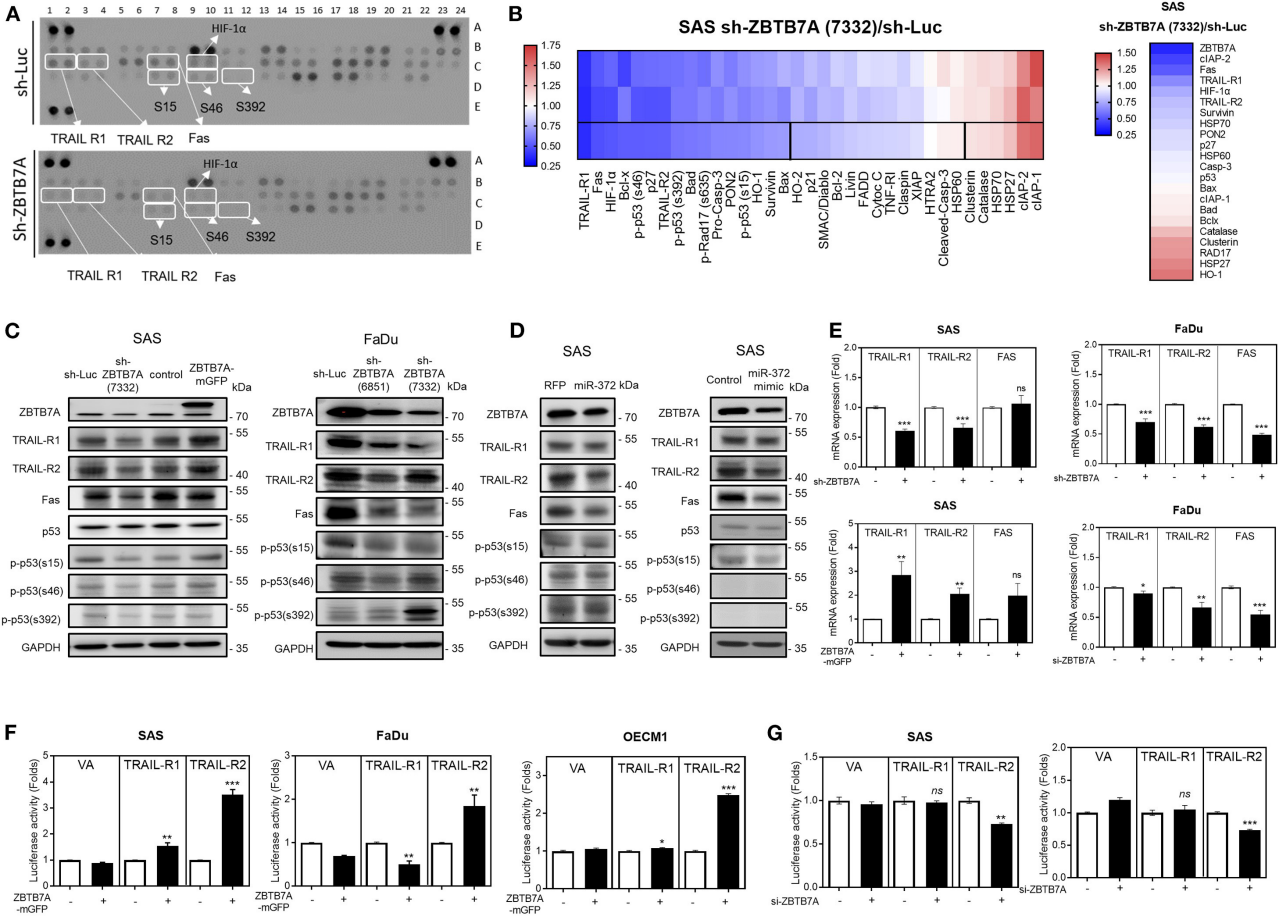
ZBTB7A expression is associated with the expression of death receptors and the phosphorylated isoforms of p53 in OSCC cells. **(A,B,D)** SAS cells. **(A)** Apoptosis array. A panel of 35 antibodies including various forms of phosphorylated p53 were measured in duplicate using this array. The ZBTB7A knockdown cell subclone and the control cells were treated with CDDP for 24 h, and then the array was used to identify changes in various apoptosis factors. Lt, a diagram of the array. White rectangles indicate duplicates exhibiting significant changes in signal following ZBTB7A knockdown. Rt, a heat-map demonstrating the changes in protein profile following ZBTB7A knockdown. The 3rd lane represents the average value of the duplicate signals in the upper two lanes. Vertical lines define the 20 proteins that are downregulated >30% (Lt region) or that are upregulated >12% (Rt region). **(B)** RNA sequencing. A heat-map showing the changes in transcript levels in the ZBTB7A subclone. The genes selected for analysis are TRAIL-R1, TRAIL-R2, Fas and the various phosphorylated isoforms of p53 and many of these were downregulated following knockdown of ZBTB7A; thus these are to be considered as ZBTB7A targets during apoptosis modulation. **(C,D)** Western blot analysis. **(C)** Lt, SAS cells, Rt, FaDu cells. Expression of TRAIL-R1, TRAIL-R2, Fas, and p53 phosphorylated at serine 15 is correlated with ZBTB7A expression (in **C**), and is inversely correlated with ZBTB7A knockdown (in **C**) and *miR-372* expression (in **D**) in OSCC cells. **(E)** qRT-PCR analysis. This shows the concordant changes in *TRAIL-R1* and *TRAIL-R2* mRNA expression in OSCC cells following ZBTB7A knockdown/expression. **(F,G)** Promoter activity assay. This shows the consistent increase and decrease in TRAIL-R2 activity that follows expression and transient knockdown of ZBTB7A in OSCC cells, respectively. *ns*, not significant; *p < 0.05; **p < 0.01; ***p < 0.001.

To unravel the downstream apoptosis factors associated with ZBTB7A, the sh-ZBTB7A (7332) subclone and sh-Luc SAS cell lines were treated with CDDP for 24 h. The apoptosis array used to analyze protein expression by these cells lines allows one to measure the alteration in expression of a number of important proteins, including TRAIL-R1, TRAIL-R2, Fas, p53 phosphorylated at serine 15, p53 phosphorylated at serine 46, p53 phosphorylated at serine 392, HIF-1α and various others (Figure 4A). RNA-sequencing was also performed to confirm the expression levels of the proteins identified as having changes in expression by the array. Decreased *TRAIL-R1, TRAIL-R2, Fas*, and *HIF-1*α expression was found in the ZBTB7A knockdown subclone compared to the control cells (Figure 4B). The expression levels of TRAIL-R1, TRAIL-R2 and Fas were also found to parallel the changes in ZBTB7A expression level in the SAS cell lines (Figure 4C). The expression of TRAIL-R1, TRAIL-R2, and Fas was decreased in FaDu cells when there was ZBTB7A knockdown. In SAS cells that were stably expressing *miR-372* or were transiently expressing *miR-372*, the expression of the above death receptors was found to be decreased (Figure 4D). The cellular level of p53 protein phosphorylated at serine 15, but not at other phosphorylation sites, was also parallel to changes in ZBTB7A expression, but these changes were in the opposite direction to the changes in *miR-372* expression (Figures 4C,D). Furthermore, ZBTB7A expression increased the levels of *TRAIL-R1, TRAIL-R2*, and *Fas* mRNA expression. In contrast, ZBTB7A knockdown decreased mRNA expression of *TRAIL-R1* and*TRAIL-R2* (Figure 4E). The influence of ZBTB7A on *HIF-1*α mRNA expression was found not to be consistent across the various OSCC cell lines (Figure S5). The JASPAR module predicted two potential ZBTB7A binding sites within the *TRAIL-R2* promoter and one binding site within the *TRAIL-R*1 promoter. On the other hand, there were no potential ZBTB7A targeting sites predicted within the *Fas* promoter. *TRAIL-R1* and *TRAIL-R2* promoter reporter constructs were generated in order to determine the influence of ZBTB7A on promoter activation of these proteins. The analysis showed that ZBTB7A expression enhanced *TRAIL-R2* promoter activity in OSCC cells (Figure 4F) and that ZBTB7A knockdown decreased *TRAIL-R2* promoter activity (Figure 4G). However, *TRAIL-R1* promoter activity was only slightly increased in SAS cells when there was exogenous ZBTB7A expression (Figures 4F,G). When these finding are taken together, it supports the hypothesis that ZBTB7A upregulates the death receptors TRAIL-R1 and TRAIL-R2, as well as expression of Fas and phospsphorylated-p53, but an increase in promoter activity was only able to affect *TRAIL-R2*. *p53* expression was not correlated with *ZBTB7A* expression in the HNSCC cohort obtained from the TCGA data-set. Finally, TRAIL-R1, TRAIL-R2, and Fas downregulation could be detected when HNSCC tumor samples were analyzed (Figure S6).

### ZBTB7A Increases Drug Sensitivity via Trans-Activation of TRAIL-R2

**Figure 5:**
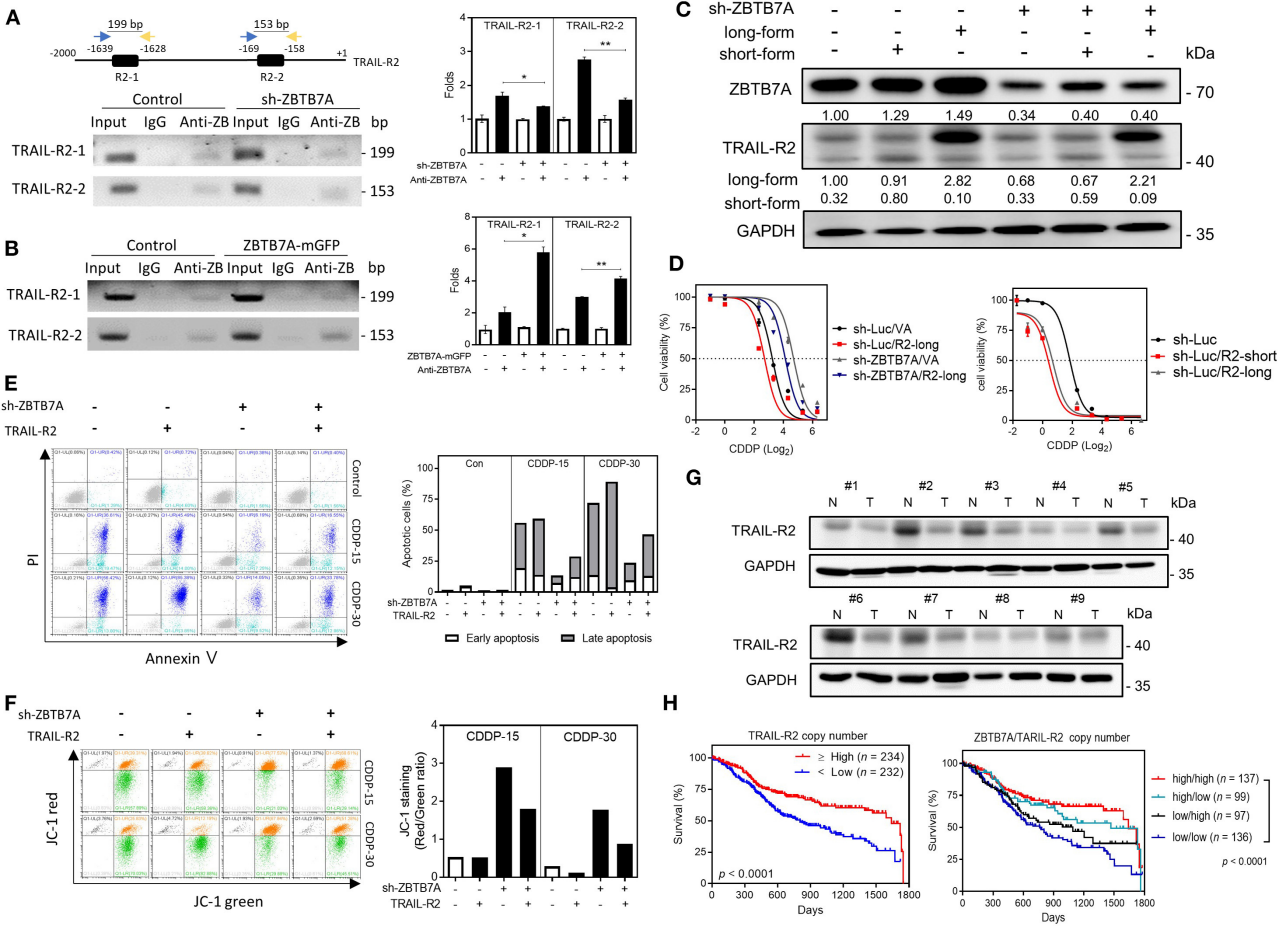
ZBTB7A sensitizes SAS cells to CDDP by trans-activation TRAIL-R2. **(A)** Lt Upper, schematic diagram depicting the ChIP strategy. R2-1 and R2-2 are predicted to be ZBTB7A binding sites in the TRAIL-R2 promoter. (**A**, Lt Lower; **B**, Lt) Representative gel electrophoresis images of the PCR analysis; these reveal the amplicons of R2-1 and R2-2 sequences using the various immunoprecipitates, namely control, ZBTB7A knockdown and ZBTB7A exogenous expression cells. (**A,B**, Rt), quantification. The analysis indicates that ZBTB7A expression increases, and ZBTB7A knockdown decreases the binding of the ZBTB7A to TRAIL-R2-1 and TRAIL-R2-2 sites, respectively. Anti-Z: anti-ZBTB7A antibody. **(C,D)** Long-form and short-form TRAIL-R2 expression, respectively. Lt, Western blot analysis confirming the expression of TRAIL-R2 in the SAS ZBTB7A knockdown cell subclone by means of TRAIL-R2 retroviral infection. Rt, CDDP sensitivity as related to expression of ZBTB7A and TRAIL-R2. **(E)** Apoptosis assay. Lt, flow cytometry diagram. Rt, quantification. The CDDP-induced apoptosis is decreased by ZBTB7A knockdown and this is reversed by TRAIL-R2 expression. **(F)** Lt, Mitochondrial membrane potential analysis. Rt, Quantification of the red/green ratio. Following 15 or 30 μM CDDP for 48 h, the increase in the red/green ratio as a result of ZBTB7A knockdown is reversed by TRAIL-R2 expression. **(G)** Western blot analysis of paired OSCC tissue samples. TRAIL-R2 expression is clearly downregulated in the OSCC tissue samples. N, NCMT; T, OSCC. **(H)** Kaplan-Meier analysis of the overall survival in relation to ZBTB7A copy number and TRAIL-R2 copy number. *ns*, not significant; *p < 0.05; **p < 0.01.

ChIP assays were conducted using immunoprecipitates from the lysates of ZBTB7A knockdown and expression cell subclones in order to explore the direct binding of ZBTB7A to the *TRAIL-R2* promoter. PCR analysis and quantitative assays revealed that ZBTB7A does specifically bind to the R2-1 and R2-2 sequences within the promoter based on the results, which showed that the yields of PCR products paralleled ZBTB7A levels (Figures 5A,B). *In silico* analysis designated the nearly absence of the complementarity between *miR-372* sequence and *TRAIL-R2* promoter sequence or transcript, excluding the possibility that *miR-372* may directly activate or target *TRAIL-R2*. A previous report has shown that the long-form and short-form of TRAIL-R2 may show differences in apoptotic induction in various tumor cells (Figure S7A) (37). There was a lack of differential expression of the long-form and the short-form when various tumor cells and normal cells were compared (Figures S7B,C). The expression of the long-form or the short-form TRAIL-R2 were correlated (Figure S7D). The control or ZBTB7A knockdown subclone was infected with retroviruses that allowed expression of the long-form or the short-form of TRAIL-R2. Expression of either the long-form or the short-form of TRAIL-R2 was able to increase CDDP sensitivity (Figures 5C,D). In addition, TRAIL-R2 expression in ZBTB7A knockdown subclone was able to increase CDDP-induced apoptotic cell death (Figure 5E), and reduce MMP (Figure 5F). Western blot analysis showed that TRAIL-R2 was downregulated in most tumor samples comparing to their normal matched tissues (Figure 5G). Analysis of the TCGA database also revealed that HNSCC tumors harboring a lower TRAIL-R2 copy number had a worse prognosis relative to their counterparts (Figure 5H). Moreover, HNSCC tumors having lower ZBTB7A expression and a lower TRAIL-R2 copy number also exhibited a worst prognosis. Collectively, these findings demonstrate that ZBTB7A increases CDDP sensitivity and apoptosis by trans-activating TRAIL-R2.

### miR-372 Depletion Is Associated With TRAIL-R2 Upregulation and Increased CDDP Sensitivity

**Figure 6:**
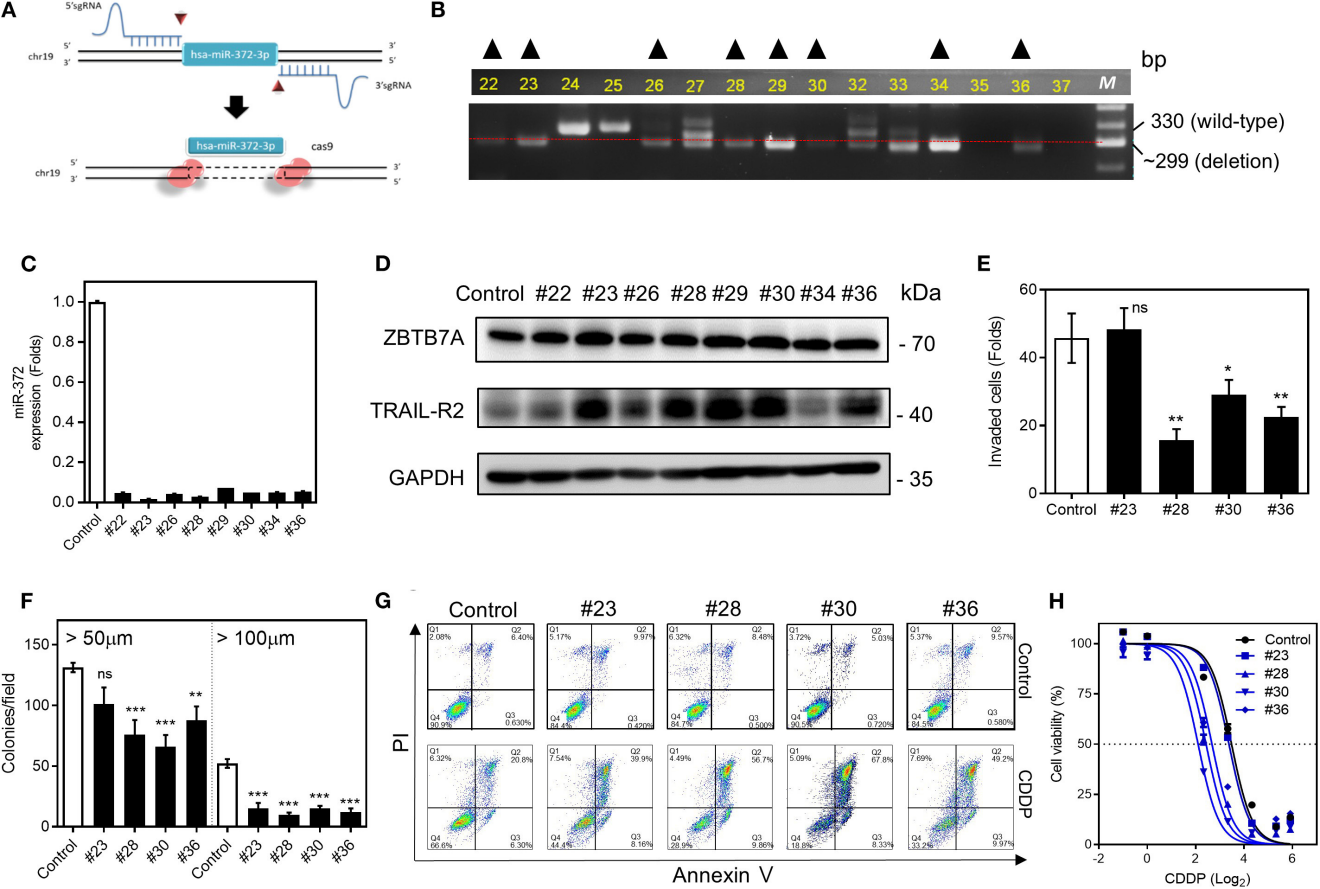
*miR-372* associated oncogenicity and CDDP resistance in SAS cells. **(A)** 5′sgRNA and 3′sgRNA are designed to delete hsa-*miR-372*-3p. **(B)** PCR analysis is used to screen the deletion cell subclones. The dotted line indicates the ~300-bp position on the gel electrophoresis image. Cell subclones exhibiting a band below the dotted line and the absence other bands (marked by triangles) are suspected to have a homozygous deletion. **(C)** qRT-PCR analysis. This reveals almost complete absence of *miR-372* expression in the sublcones selected in **(B)**. **(D)** Western blot analysis revealing the upregulation of ZBTB7A, TRAIL-R1, and TRAIL-R2 expression in nearly all subclones except for subclone #34. **(E,F)** Invasion assay and anchorage-independent colony formation assay, respectively. The assays show the general decrease of these properties in the deleted subclones that are tested. **(G)** Flow cytometry analysis to detect apoptosis. The CDDP induced apoptosis is higher in deleted subclones relative to the control cells. **(H)** Cell viability assay. The *miR-372* deleted cell subclones exhibit higher sensitivity to CDDP. *ns*, not significant; *p < 0.05; **p < 0.01; ***p < 0.001.

Using the Crispr/Cas 9 gene editing strategy (Figure 6A), we have been able to establish multiple cell subclones using SAS cells (Figure 6B) that showed lower levels of *miR-372* expression (Figure 6C). Increases in both ZBTB7A and TRAIL-R2 expression could be noted in the vast majority of the deleted subclones analyzed (Figure 6D). The invasion and colony formation competences of these subclones were usually decreased following *miR-372* depletion (Figures 6E,F). Apoptosis, when induced by CDDP, was more obvious in these deleted subclones compared to the controls (Figure 6G). In addition, *miR-372* depletion also increased the sensitivity to CDDP (Figure 6H). Based on the above findings, deletion of *miR-372* would seem to be a potential strategy to block OSCC drug resistance.

## Discussion

OSCC is one of the most common cancers worldwide. Although there have been recent advances in the diagnosis and treatment of OSCC, drug resistance remains a challenging issue when treating OSCC (1). The five-year survival rate of OSCC has remained low over the past few decades and therefore new strategies regarding HNSCC therapy, especially those that are able to increase the efficacy of conventional chemotherapy, are urgently needed. *miR-372* is a hypoxia-inducible miRNA, expression of which is frequently changed in cancers (5). We previously have discovered that p62 is a target of *miR-372* and that the *miR-372*-p62 axis is able to modulate reactive oxidative species (ROS) and promote the OSCC neoplastic process (4). Expression of *miR-372* is known to be correlated with tumor progression and stage in both tissue and serum sample (4, 15). This study provides further clues regarding the involvement of *miR-372* and various new downstream effectors in relation to the modulation of OSCC oncogenicity and drug resistance.

The role of ZBTB7A in tumor pathogenesis are controversial (18, 19, 21, 22). Although lower expression of ZBTB7A has been shown to occur in OSCC samples (23), its fundamental role in OSCC pathogenesis has remained unclear. This study has identified that downregulation of ZBTB7A occurs in OSCC and that this protein acts as a suppressor of OSCC. ZBTB7A is localized at chromosome 19p13.3, a locus is known to be deleted in HNSCC (2), thus gene copy number decrease is one plausible cause of ZBTB7A downregulation in HNSCC. Interestingly, the association between a low ZBTB7A copy number and poorer survival of HNSCC patients can be seen when the in TCGA data-set is analyzed. As *miR-372* is able to target ZBTB7A, which is an oncogenic suppressor, we further suggest that there is a reverse correlation lying between *miR-372* expression and ZBTB7A expression. The presence of an association between ZBTB7A downregulation and poor patient survival further support for the importance of ZBTB7A alterations in HNSCC. In previous studies, *miR-20a, miR-100* and *miR-125a* have been shown to be suppressor miRNAs that are able to reduce expression of the ZBTB7A proto-oncogene in tumors (26–28). However, it has been found that *miR-106b* targeting of ZBTB7A increases the survival of hepatocellular carcinoma cells (25). This study further identifies that ZBTB7A is also under the post-transcriptional control of *miR-372* in addition to there being gene copy loss during oral tumorigenesis. Since *miR-372* is a prognostic determinant of a wide panel of malignancies (8, 11, 13), the *miR-372*-ZBTB7A axis would seem likely to be a therapeutic target when treating such malignancies.

The findings regarding the CDDP resistance and taxol resistance by the various cell subclones suggests the involvement of *miR-372*-ZBTB7A in drug resistance. We have further identified a linkage between ZBTB7A and apoptotic cell death as a result of CDDP and taxol treatment. Specifically, an inhibition of autophagy brings about an increase in CDDP toxicity (45), and this study also indicates that induction of autophagy increases CDDP resistance in OSCC cells. Caloric restriction brings about low levels of apoptosis, high levels of autophagy and ZBTB7A downregulation when the HCT116 3D model cell model is investigated (46). *miR-372* is hypoxia inducible (5), and it is known to enhance autophagy in nerve cells (47). Nevertheless, whether, *miR-372* is involved in autophagy induction, which then modulates the inhibition of apoptosis that results from ZBTB7A targeting, needs further investigation.

Ferroptosis has been shown to sensitize HNSCC cells to CDDP treatment (48). However, our preliminary assays did not show a decrease in drug-induced apoptosis following blockage of ferroptosis. *p53* mutations frequently reside in malignancies including HNSCC (49). *p53*, either the its wild type form or its mutant form, are widely considered to be crucial factors in CDDP induced apoptosis (50). However, this study was unable to address the interplay of ZBTBA and p53 in the responses to CDDP. We found that the level of ZBTB7A was correlated in OSCC cells with the abundance of the isoform of p53 that is phosphorylated at serine-15. Serine-15 phosphorylation is highly associated with p53 activity during transcriptional regulation (51). Although a study has shown that ZBTB7A is able to represses ARF, and this might lead to indirect p53 inactivation (19), it also seems likely that ZBTB7A is able to regulate p53 activity by modulating the protein's phosphorylation state.

The death receptors TRAIL-R1, TRAIL-R2, and Fas were identified as downstream factors of ZBTB7A. However, ZBTB7A is only able to trans-activates TRAIL-R2 among these death receptors; it does this via an increase in promoter activity and mRNA expression. It has been reported that TRAIL-R2 selective ligands are superior to TRAIL-R1 selective ligands when triggering apoptosis in colorectal and pancreatic cancer cells (52). Recent studies have shown that ZBTB7A modulates apoptosis and chemosensitivity via a range of different mechanisms across diverse malignancies (24, 53). The present study has identified a new function of ZBTB7A, namely the activation TRAIL-R2, which then results in a triggering of apoptosis in OSCC. Intriguingly, although ZBTB7A has been demonstrated as a transcription repressor in many reports (18, 19, 22, 54), a recent study has specified ZBTB7A as both a transcription activator and a repressor, which modulates the differential regulation of human beta-like globin expression during the developmental process (55). Moreover, ZBTB7A has been known for a co-operator of transcription factors, such as NFκB or HIF, to induce gene expression (18). Therefore, the functional complex associating with of ZBTB7A in transactivating TRAIL-R2 needs further elucidation. The mechanisms regulating TRAIL-R1, Fas, and p53 phosphorylation also remain elusive. The regulation of death receptors is known to involve extrinsic apoptosis pathways, while the intrinsic pathway is dependent on involvement of the mitochondria. Mitochondrial ROS genesis or fission, both of which facilitate apoptosis, are correlated with CDDP sensitivity (44, 56). Although our array analysis did give some limited insights into changes of intrinsic regulation that are mediated by ZBTB7A during apoptotic induction, our experiments identify that ZBTB7A downregulation ablates the CDDP-induced mitochondrial collapse. Despite that the TRAIL signaling may activate elements to enhance intrinsic pathway (57), detailed investigations are now required in order to profile the intrinsic factors that are able to be regulated by ZBTB7A.

We also investigate the involvement of the TRAIL-R2 isoforms in the above processes and our findings indicate that both isoforms are expressed in the cells we investigate, and both bring about similar levels of apoptotic induction in OSCC cells. Although these findings are in conflict with previous findings using lung cancer cells (37), whether differential TRAIL-R2 functionality is a global scenario in malignancies needs more study. Furthermore, the expression of TRAIL-R2 in ZBTB7A knockdown subclones is unable to completely rescue the apoptotic repression secondary to ZBTB7A silencing, which suggests that ZBTB7A may have a number of potent impacts other than TRAIL-R2 during the modulation of apoptosis and it seem likely that this will occur via other molecules.

Obvious downregulation of TRAIL-R2 is found in our tumor samples. As the TRAIL-R family is localized at 8p21.3, a locus that is prone to deletion in OSCC (2), our findings of a worse prognosis when HNSCC tumors have a lower TRAIL-R2 copy number using the TCGA cohort confirms the importance of TRAIL-R2 in tumor abrogation. Furthermore, since the loss of both 8p21.3 and 19p13.3 might also be present in this OSCC cohort (2), such concordant copy loss of both the ZBTB7A gene and the TRAIL-R2 gene may help to create this worsening in HNSCC survival. Tumors undergo EMT may acquire N-cadherin expression. A decrease in TRAIL-R2 expression would also contribute to increased N-cadherin expression (38). Since the EMT is highly associated with drug resistance (58), it is likely that the ZBTB7A-TRAIL-R2 axis may also attenuate EMT-associated drug resistance. Since disruptions of the *miR-371*-*miR-373* cluster and the chromosome 19q13 locus are rather common in malignancies (4, 6, 8, 10, 12–14), genome-wide studies should help with a more comprehensive understanding of how aberrations in the *miR-372*-ZBTB7A-TRAIL-R2 cascade affect oncogenicity.

TRAIL has been found to increase the tumoricidal effects of CDDP against HNSCC xenografts (59). This study provides clues demonstrating that *miR-372*-ZBTB7A cascade modulates TRAIL-R2 expression in sensitizing OSCC cells for CDDP-induced apoptosis. DcR-1 and DcR-2 are able to inhibit the TRAIL pathway (29, 37). Since DcR-2 and TRAIL-R2 are co-expressed in HNSCC tumors (60), the efficacy of TRAIL-mediated HNSCC therapy needs validation (34, 35). As the resistance to TRAIL seems to exist in most tumor cells (33–35), the trials of newly developed anti-TRAIL-R2-drug conjugate reagents coupling with anti-*miR-372* strategies could be elaborated as a potent therapeutic approach with the aim of ameliorating oncogenesis (34–36).

Our results demonstrate that ZBTB7A functions as a tumor suppressor in OSCC. ZBTB7A expression increases sensitivity to CDDP by direct binding to TRAIL-R2 promoter allowing transactivation. The *miR-372*-ZBTB7A-TRAIL-R2 regulatory axis, once verified, may help with prognosis prediction. In addition, *miR-372* depletion or ZBTB7A upregulation could be used as a strategy to suppress tumorigenesis or to sensitize tumors to therapy via TRAIL activation.

## Data Availability Statement

Publicly available datasets were analyzed in this study, these can be found in the NCBI Gene Expression Omnibus (GSE143273).

## Ethics Statement

This work is approval by the IRB committee of Taipei Veterans General Hospital (Approval No. 2017-07-023AC) and IRB committee of Mackay Memorial Hospital with approval numbers 11MMHIS026 and 17MMHIS164. Tissues are collected after obtaining written informed consent.

## Consent for Publication

All authors have approved the publication of this article.

## Author Contributions

L-YY, S-CL, and K-WC: study design, data analysis, and manuscript writing. C-CY, S-YK, and C-JL: tissue collection and clinical assessment. L-YY, H-LW, and Y-FC: experiment conduction.

### Conflict of Interest

The authors declare that the research was conducted in the absence of any commercial or financial relationships that could be construed as a potential conflict of interest.

## Abbreviations

BrdU: 5-bromo-2′-deoxyuridine
CDDP: cisplatin
ChIP: Chromatin immunoprecipitation
DMOG: dimethyloxaloylglycine
EMT: epithelial-mesenchymal transition
FPKM: fragments per Kb of transcript per million mapped reads
HNSCC: head and neck squamous cell carcinoma
LATS2: large tumor suppressor kinase 2
miRNA: microRNA
MMP: mitochondrial membrane potential
Mut: mutation
NCMT: non-cancerous matched tissue
OSCC: oral squamous cell carcinoma
qRT-PCR: quantitative reverse transcription polymerase chain reaction
TCGA: The Cancer Genome Atlas
TRAIL: tumor necrosis factor related apoptosis-inducing ligand
TRAIL-R1: tumor necrosis factor related apoptosis-inducing ligand receptor 1
TRAIL-R2: tumor necrosis factor related apoptosis-inducing ligand receptor 2
WT: wild type
ZBTB7A: zinc finger and BTB domain containing 7A.
