# Supplementary material for: The miR-372-ZBTB7A Oncogenic Axis Suppresses TRAIL-R2 Associated Drug Sensitivity in Oral Carcinoma
Source: Front Oncol. 2020 Jan 31;10:47. doi: 10.3389/fonc.2020.00047 (PMC7005910; doi:10.3389/fonc.2020.00047)
Supplement: Supplementary file 1 [file Data_Sheet_1.docx]

**Supplementary Tables:**

**Table S1. The STR analysis of cells used in this study**

| **Name** | **OC3** | **FaDu** | **SAS** | **HSC3** | **OECM1** | **NOK** | **DPC14** | **PDL9-1** | **PDL-YM1** | **iPDL2** |
| --- | --- | --- | --- | --- | --- | --- | --- | --- | --- | --- |
| TH01 | 7,10 | 8,8 | 6,7 | 6,9.3 | 9,9 | 7, 9 | 7,7 | 9,9 | 7,10 | 9 |
| TPOX | 9,11 | 11,11 | 11,12 | 8,8 | 8,8 | 8, 11 | 8,8 | 8,11 | 11,12 | 9,10 |
| vWA | 14,17 | 15,17 | 14,17 | 14,17 | 14,16 | 17 | 14,16 | 18,18 | 14,18 | 17,18 |
| Amelogenin | X,Y | X,Y | X,X | X,Y | X,Y | XY | X,Y | XX | XX | XX |
| CSF1PO | 10,12 | 12,12 | 11,11 | 11,11 | 12,12 | 9, 12 | 13,13 | 10,12 | 10,11 | 12 |
| D16S539 | 9,11 | 11,11 | 12,13 | 9,9 | 10,12 | 9, 11 | 9,11 | 11,12 | 9,10 | 9,12 |
| D7S820 | 9,11 | 11,12 | 11,12 | 13,13 | 8,11 | 11 | 11,12 | 10,11 | 10,11 | 10,11 |
| D13S317 | 10,11 | 8,9 | 10,12 | 12,12 | 10,10 | 8, 12 | 9,11 | 8,11 | 8,9 | 8,8 |
| D5S818 | 11,12 | 12,12 | 9,9 | 11,13 | 10,11 | 11, 12 | 10,11 | 11,12 | 11,13 | 12,13 |

**Table S2. siRNAs and control oligonucleotide used in this study**

| **siRNA** | **Supplier** | **Cat. No.** |
| --- | --- | --- |
| si-Scr | Santa Cruz Biotech | sc-37007 |
| si-ZBTB7A | Santa Cruz Biotech | sc-44574 |
| si-TRAIL-R1 | Santa Cruz Biotech | sc-35218 |
| si-TRAIL-R2 | Santa Cruz Biotech | sc-40237 |

**Table S3. The TaqMan probes used in this study**

| **probe** | **Supplier** | **Cat. No.** |
| --- | --- | --- |
| miR-372 | Applied biosystems | 000560 |
| miR-373 | Applied biosystems | 000561 |
| ZBTB7A | Applied biosystems | Hs00252415_s1 |
| TRAIL-R1 | Applied biosystems | Hs00269492_m1 |
| TRAIL-R2 | Applied biosystems | Hs00366278_m1 |
| FAS | Applied biosystems | Hs00236330_m1 |
| HIF1α | Applied biosystems | Hs00153153_m1 |

**Table S4. The primary antibodies used in this study**

| **Antibody** | **MW** | **Host** | **Dilution** | **Supplier** | **Cat. No.** |
| --- | --- | --- | --- | --- | --- |
| GAPDH | 37 | mouse | 1: 10000 | Santa Cruz Biotech. | sc32233 |
| ZBTB7A | 72 | hamster | 1:2000 | Santa Cruz Biotech. | sc33683 |
| ZBTB7A* | 72 | rabbit | 10 μg | BETHYL | A300549-A |
| TRAIL-R1 | 50 | goat | 1:500 | R&D Systems | AF347 |
| TRAIL-R2 | 45 | goat | 1:500 | R&D Systems | AF631 |
| FAS | 50 | goat | 1:500 | R&D Systems | AF326 |
| p53 | 53 | mouse | 1:1000 | Santa Cruz Biotech | sc-47698 |
| p-p53(s15) | 53 | rabbit | 1:1000 | Cell Signaling Tech. | 9284S |
| p-p53(s46) | 53 | rabbit | 1:1000 | Cell Signaling Tech. | 2521S |
| p-p53(s392) | 53 | rabbit | 1:1000 | Cell Signaling Tech. | 9281S |
| BrdU |  | mouse |  | Santa Cruz Biotech. | sc32323 |

* For ChIP assay

**Table S5. The secondary antibodies used in this study.**

| **Antibody** | **Dilution** | **Supplier** | **Cat. No.** |
| --- | --- | --- | --- |
| anti-goat | 1:1000 | Millipore | AP106P |
| anti-hamster | 1:1000 | Abcam | ab5745 |
| anti-mouse | 1:1000 | Millipore | AP124P |
| anti-rabbit | 1:1000 | Millipore | AP132P |

**Table S6. Clinical parameters of OSCC tumor samples**

| *n = 61* | |
| --- | --- |
| Age (mean ± SE) | 55.4±11.6 |
| Gender (Male/Female) | (54/7) |
| T1-3 | 23 |
| T4 | 38 |
| N0 | 39 |
| N+ | 22 |
| Stage I-III | 20 |
| Stage IV | 41 |
| Relapse or new lesion | 6 |
| Disease-free | 0 |
| Death | 11 |
| Alive | 50 |

**Table S7. Primers used in this study**

| **Construct** |  | **Sequences (5’ - 3’)** | **Amplicon (bp)** |
| --- | --- | --- | --- |
| **Reporter assay** | | | |
| WT | F | cccGAGCTCCTGGGGTGGGCTTTTAATTT | 636 |
|  | R | cccAAGCTTGGGTTCCTCTGATCGTGACT |  |
| Mut1 | F | CTTCCTCAGGAGGTACCCTAAGGGAGG |  |
|  | R | CCTCCCTTAGGGTACCTCCTGAGGAAG |  |
| Mut2 | F | TTCCTTCACGAGGTACCCAACGGCG |  |
|  | R | CGCCGGTTGGGTACCTCGTGAAGGAA |  |
| Mut3 | F | CCCAAATTTTTAAGCTCGAGTTTAGATT |  |
|  | R | AATCTAAACTCGAGCTTAAAAATTTGGG |  |
| ***miR-372* deletion** | | | |
| sgRNA | 5’ | CACTATTCTGATGTCCAAG |  |
|  | 3’ | GTGACGCCCATATCAACGGA |  |
| Identification | F | AAGACTCAACCTGCGGAGAA | Wild-type: 330  Deletion: ~299 |
|  | R | TACAGCCAGACGCTGTAAGG |  |
| **Promoter reporter constructs** | | | |
| TRAIL-R1 | F | ccccACGCGTTGGGTAGTAGGGGGAGGACT | 1599 |
|  | R | ccccAGATCTACTTCGCATTCGGAGTTCAG |  |
| TRAIL-R2 | F | ccccACGCGTGGCAATGAGGGGTTTAGCA | 1938 |
|  | R | ccccAGATCTCAACTGCAAATTCCACCACA |  |
| **ChIP assay** | | | |
| R2-1 | F | CATGCCTGAGCCTCTCACC | 199 |
|  | R | GTGGAGCTGCCTGCCAGT |  |
| R2-2 | F | TGGAAAGCGGACTCTGAACC | 153 |
|  | R | CGGGCTGTGGTTTGTTTCTG |  |
| **TRAIL-R2 expression** | | | |
| TRAIL-R2 CDS | F | cccTACGTACTGCGCCCACAAAATACAC | 1483 or 1396 |
|  | R | cccGTCGACAGTCCAGTTGGGCTTTTTCC |  |
| Sequencing primer | F1 | CTTTATCCAGCCCTCAC |  |
|  | F2 | CCACGGGCCTGAGAGACTAT |  |
|  | F3 | CGGGAAGAAGATTCTCCTGA |  |
|  | R1 | CAGGTCGTTGTGAGCTTCTG |  |
|  | R2 | ACCCTAACTGACATTCC |  |
| **TRAIL-R2 isoform certification** | | | |
|  | F | GATGGTCAAGGTCGGTGATT | Long form: 303  Short form : 216 |
|  | R | CGTTGTGAGCTTCTGTCCAC |  |

**Table S8. shRNA constructs used in this study**

|  | **Clone ID** |
| --- | --- |
| sh-Luc | TRCN0000072249 |
| sh-ZBTB7A(6851) | TRCN0000136851 |
| sh-ZBTB7A(7332) | TRCN0000137332 |

**Supplementary Figures**

**Figure S1. Comparison of *ZBTB7A* mRNA expression between SAS and normal cells.** Compared to the SAS cells, all normal mesenchymal cells, namely dental pulp cells, DPC14 cells, periodontal ligament cells, PDL9-1, PDL-YM1 and iPDL2, have significantly higher *ZBTB7A* mRNA expression levels. ***, *p* < 0.001.

**Figure S2. Analysis across tumor stage and clinicopathological parameters based on the TCGA HNSCC data-set.** Tumor stage is correlated with HPV status, with p16 expression, with pathological grade, with gender and with ZBTB7A expression. *γ,* correlation coefficient. *, *p* <0.05; ***, *p* <0.001.

**Figure S3. Schematic diagram of the ZBTB7A 3’UTR reporter constructs.** (A - C), Mut1 to Mut3, respectively. Blue boxes indicate the mutant sequences. WT, wild-type reporter.

**Figure S4. ZBTB7A expression affects the cell cycle and apoptosis in SAS cells.** (A*)* Representative cell cycle analysis of ZBTB7A knockdown or exogenous expression cell subclones using flow cytometry. (B) A summary of the cell cycle profiles based on a triplicate analysis. ZBTB7A knockdown decreases the G0/G1 phase and increase the S and G2/M phases. (C) Summary of Sub-G1 phase. This seems to suggest that ZBTB7A may also change the Sub-G1 phase. (D) Cell cycle analysis of synchronized cells. To more specifically address the influences of ZBTB7A on the cell cycle and cell apoptosis, the cells are treated with 2 mM hydroxyurea for 24 h to arrest the cell cycle. Cycle phases at the subsequent time points after the release from arrest are shown. The analysis shows a decrease in the Sub-G1, G0/G1 and S fractions together with an increase in G2/M fraction for the ZBTB7A knockdown subclone after synchronized growth for 24 h. An increase in the Sub-G1 fraction and a decrease in the G2/M fraction can also be seem when the ZBTB7A exogenous expression subclone investigated. The cell cycle data for this experiment at 24 h is summarized in Figure 2*d*. HU, hydroxyurea.

**Figure S5. *HIF*αmRNA expression following ZBTB7A knockdown and expression.** (A) SAS cells. (B). FaDu cells. *ns*, not significant; **, *p* <0.01.

**Figure S6. Analysis of p53 and various death receptors using the TCGA HNSCC data-set.** (A) Lt, correlation analysis. X-axis, Z-score of p53. Y-axis, Z-score of ZBTB7A. Rt, ZBTB7A expression (Z-score in Y-axis) as related to p53 expression level. ZBTB7A expression shows no correlation with p53 expression. *ns*, not significant. (B) Lt, TRAIL-R1, TRAIL-R2 and Fas expression in the HNSCC samples. Y-axis, Z-score. Downregulation of TRAIL-R1, TRAIL-R2 and Fas expression is present in the HNSCC tumor samples. Rt, TRAIL-R2 copy number in the HNSCC samples. Both dot plot (Lt panel) and Box-and-Whiskers plot (Rt panel) of TRAIL-R2 copy number are shown. Median value marked by dot-line is used as cut-offs to define high *vs* low. +, mean value.

**Figure S7. Both the long- and the short- TRAIL-R2 isoforms contribute to CDDP induced cell death using SAS cells.** (A) A diagram illustrating the difference between the TRAIL-R2 long-form and TRAIL-R2 short-form. Alternative pre-mRNA splicing causes 87-bp shortness in mRNA length and the loss of 29 amino acids in extracellular domain of short-form. (B) Expression of the TRAIL-R2 isoforms in various cell lines. Lt, gel electrophoresis images of the RT-PCR analysis showing the long-form amplicon (the 303-bp band) and short-form amplicon (the 216-bp band). Left panel, NOK and OSCC cell lines. Right panel, normal oral mesenchymal cells. Numbers, the long-form and short-form signals. (C*)* Comparison of TRAIL-R2 isoform expression across normal cells and OSCC cells. *ns*, not significant. (D) Correlation analysis between TRAIL-R2 long-form and short-form expression. It shows a correlation in the expression of these two isoforms in cells.
