# Supplementary figures and images for: The miR-372-ZBTB7A Oncogenic Axis Suppresses TRAIL-R2 Associated Drug Sensitivity in Oral Carcinoma
Source: Front Oncol. 2020 Jan 31;10:47. doi: 10.3389/fonc.2020.00047 (PMC7005910; doi:10.3389/fonc.2020.00047)

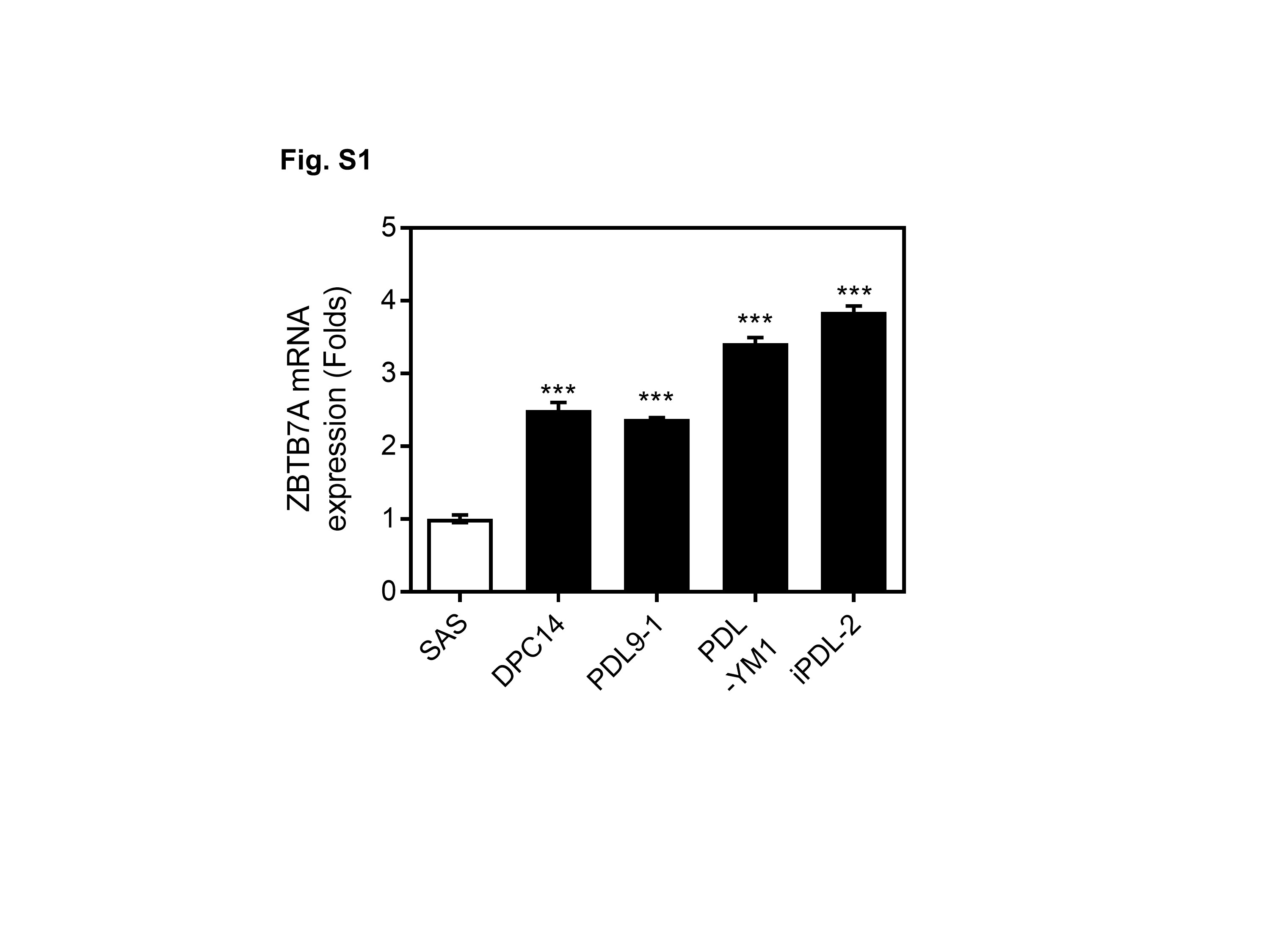

Supplement: Supplementary file 2 [file Image_1.JPEG]

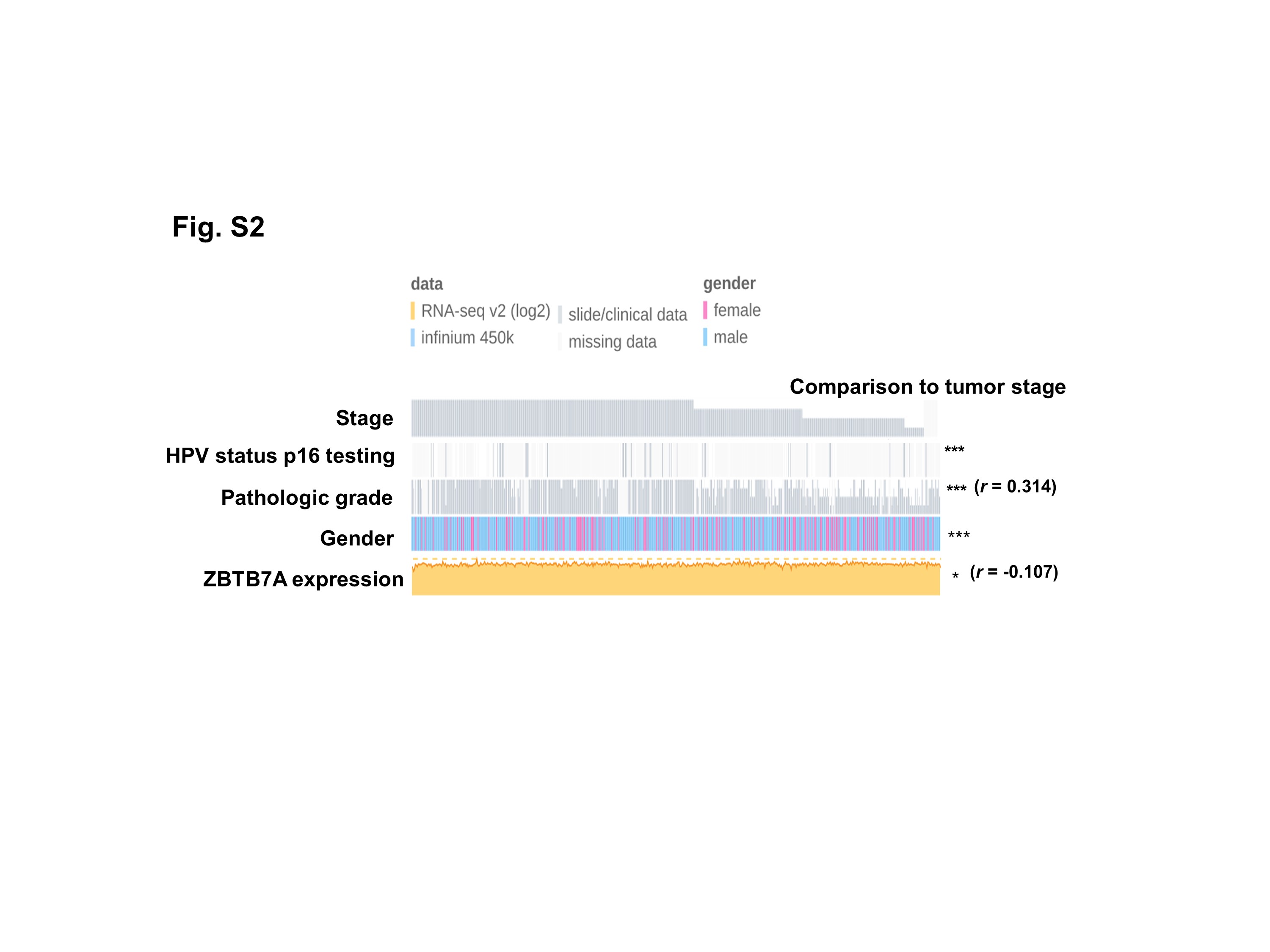

Supplement: Supplementary file 3 [file Image_2.JPEG]

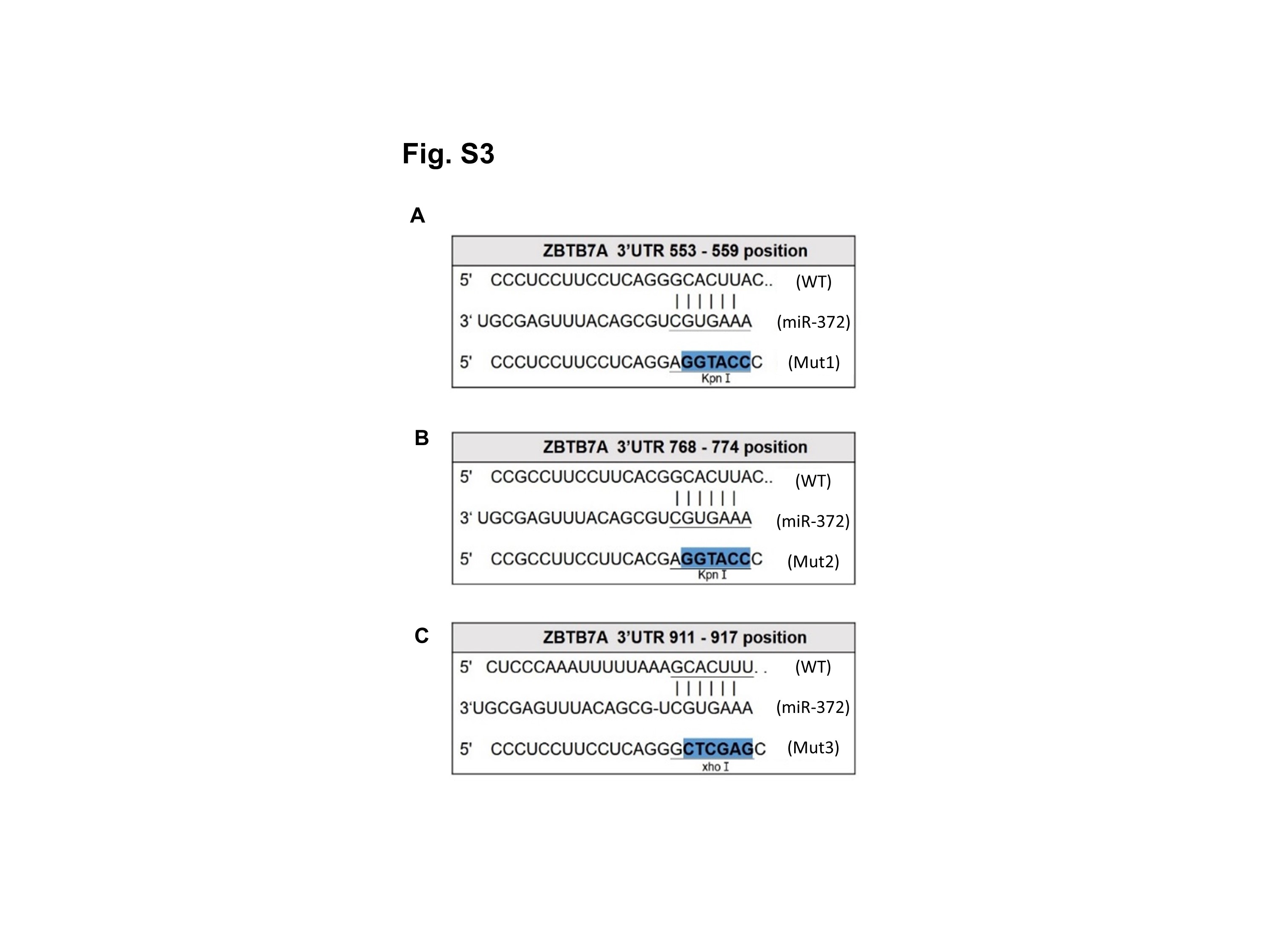

Supplement: Supplementary file 4 [file Image_3.JPEG]

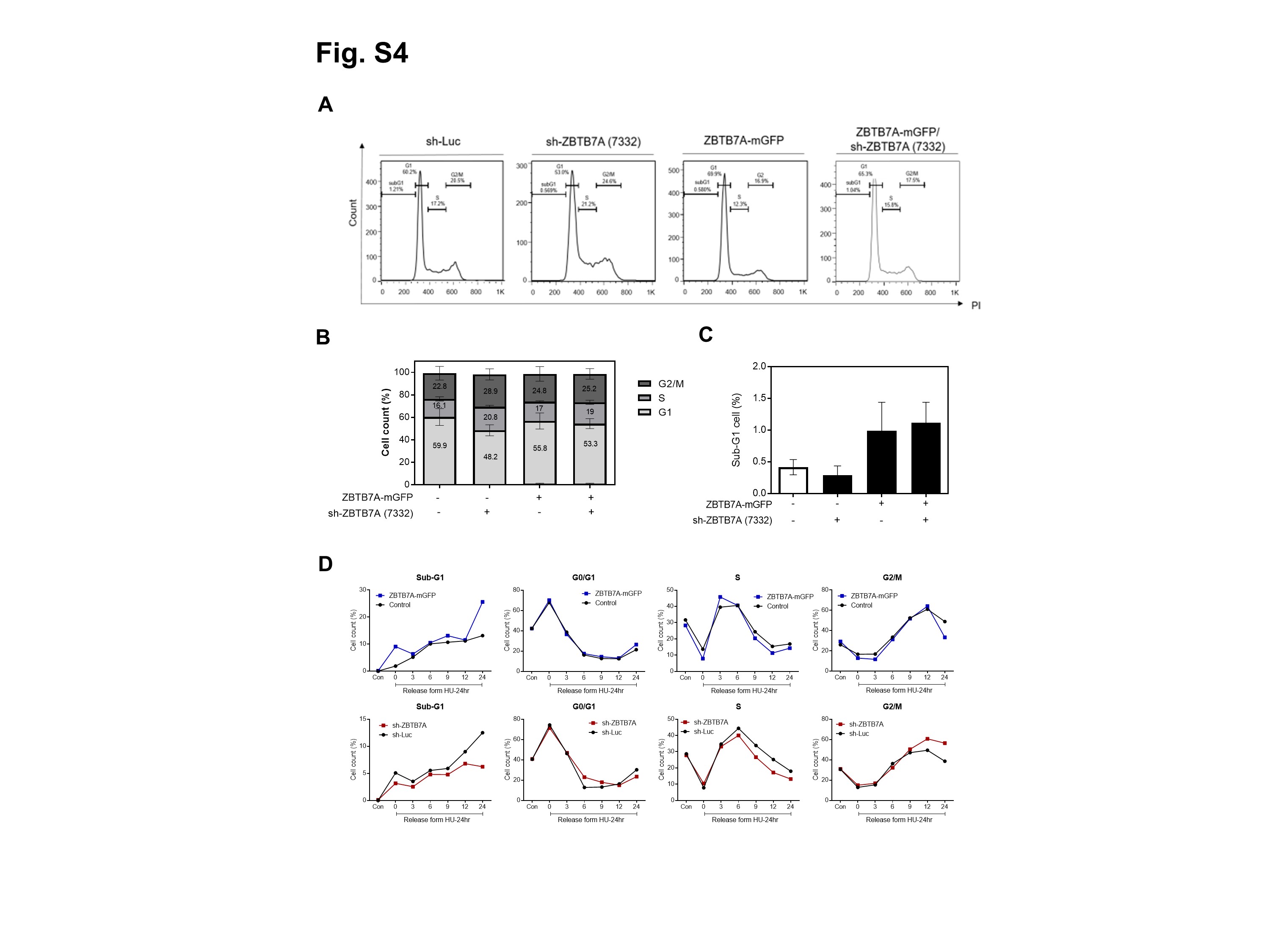

Supplement: Supplementary file 5 [file Image_4.JPEG]

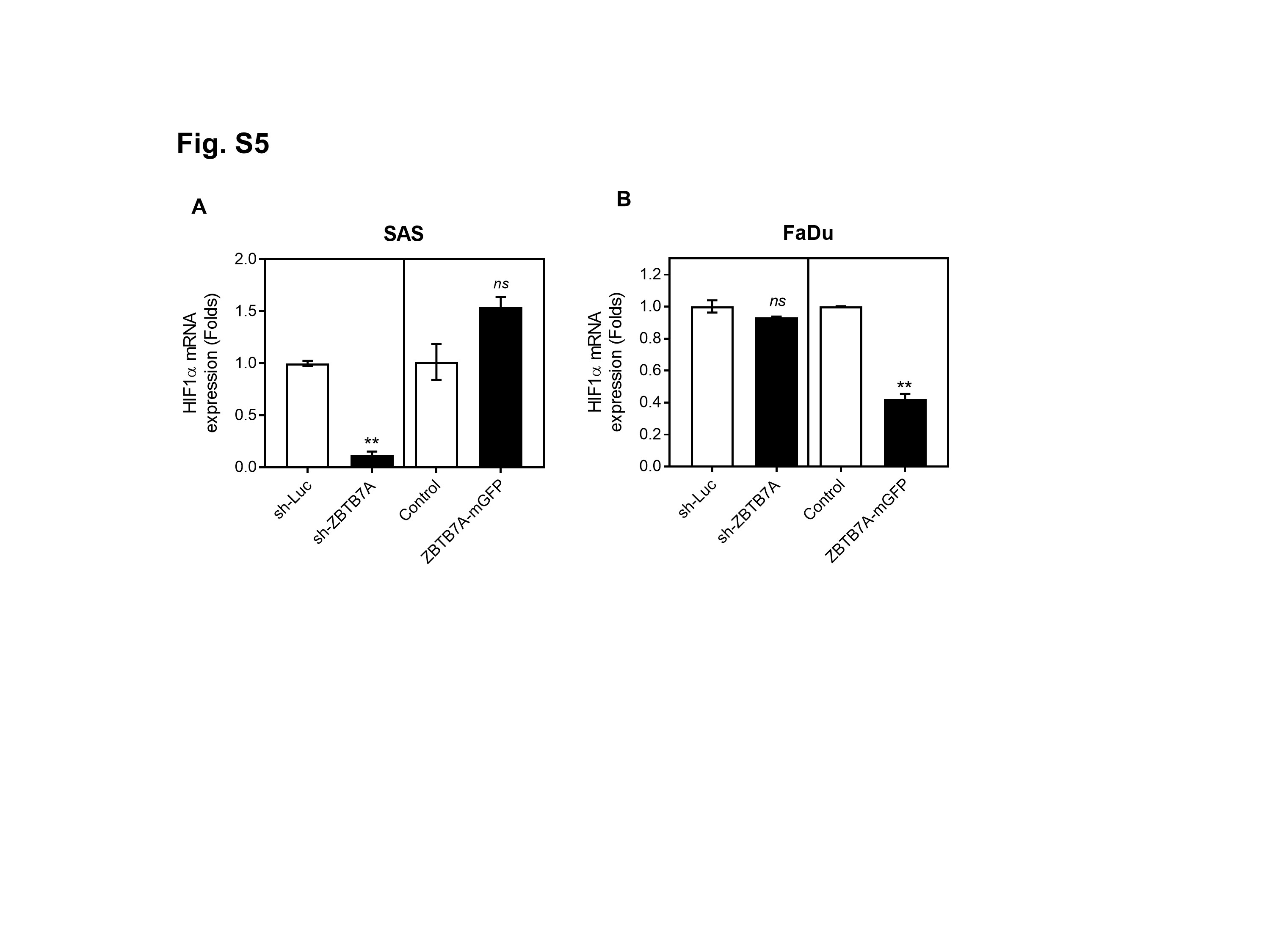

Supplement: Supplementary file 6 [file Image_5.JPEG]

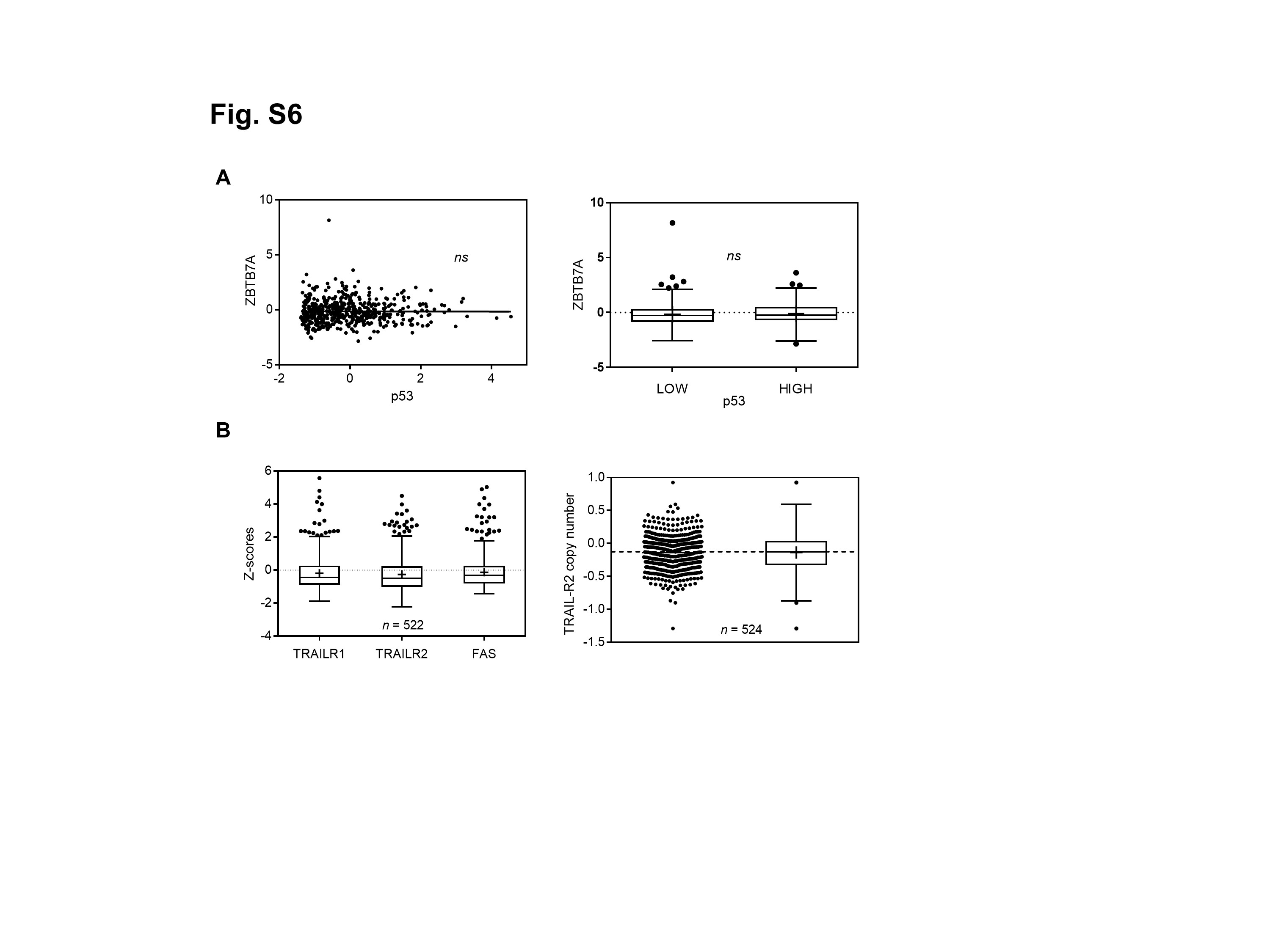

Supplement: Supplementary file 7 [file Image_6.JPEG]

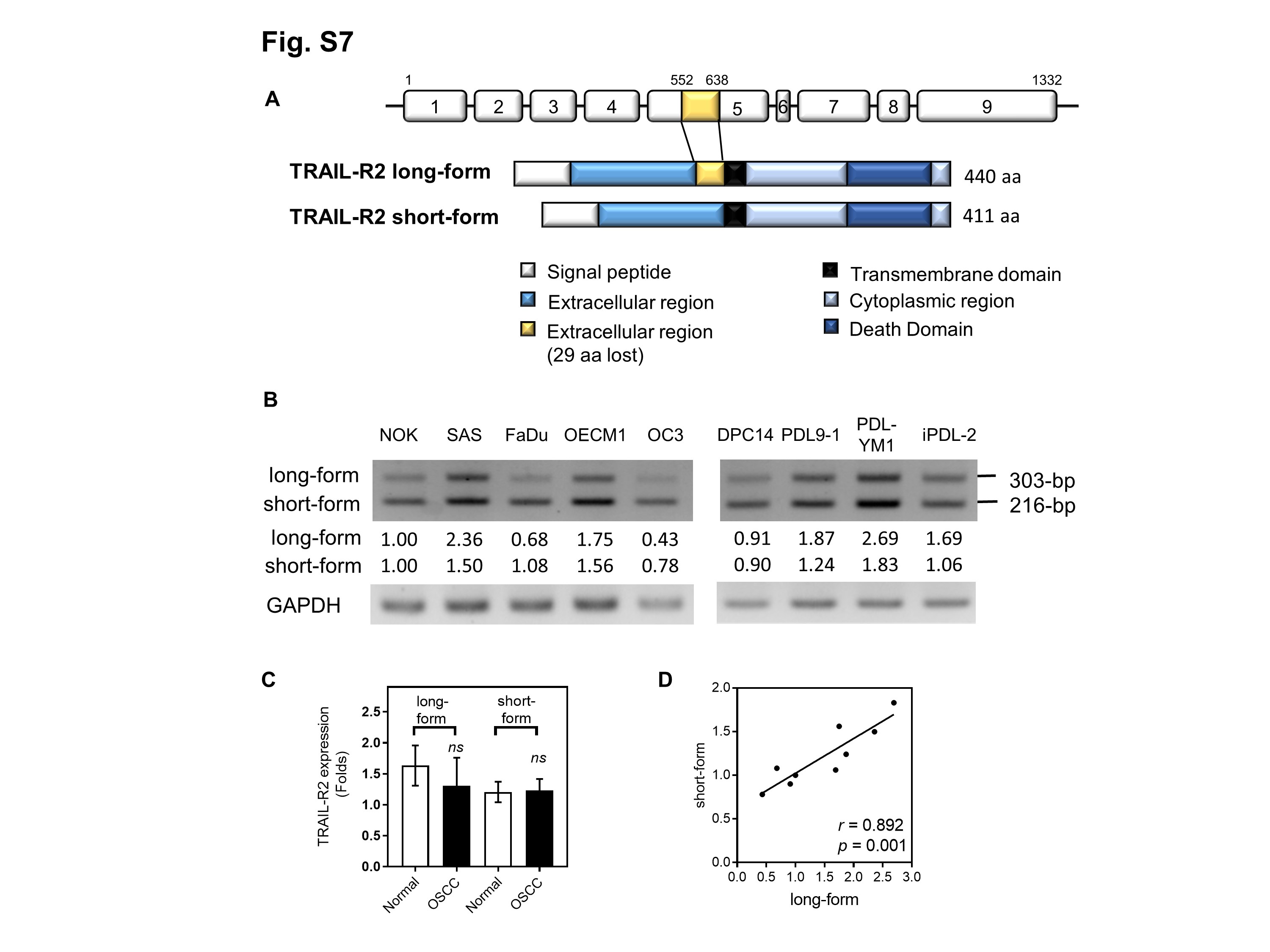

Supplement: Supplementary file 8 [file Image_7.JPEG]
